# Morphological diversity in the honeyeater hyolingual apparatus and its relationship with nectarivory

**DOI:** 10.1371/journal.pone.0338219

**Published:** 2025-12-04

**Authors:** Amanda E. Hewes, Cassandra Fieldson, Maude W. Baldwin, William A. Buttemer, Alejandro Rico-Guevara

**Affiliations:** 1 Department of Biology, University of Washington, Life Sciences Building, Seattle, Washington, United States of America; 2 Burke Museum of Natural History and Culture, Seattle, Washington, United States of America; 3 Max Planck Institute for Biological Intelligence, Eberhard-Gwinner-Straße, Seewiesen, Germany; 4 Centre for Integrative Ecology, Deakin University, Geelong, Victoria, Australia; CONICET: Consejo Nacional de Investigaciones Cientificas y Tecnicas, ARGENTINA

## Abstract

Honeyeaters (Aves, Meliphagidae) are a speciose clade of nectarivorous birds, and there is immense diversity in the degree to which different species within the family rely on nectar. Honeyeater tongues are commonly described as similar to a paintbrush, with this morphology being interpreted as an adaptation for increasing nectar extraction efficiency. However, there has been limited work documenting the degree of interspecific diversity in tongue morphology across the family or the extent to which such diversity correlates with dependance on nectar. This information is also lacking for the hyoid bones, the structures responsible for moving the tongue in and out of the mouth. We aimed to fill this knowledge gap by examining honeyeater tongues and hyoids from across the family. We found that there are six distinct tongue types across the Meliphagidae, and that certain genera such as *Acanthorhynchus* and *Phylidonyris* have a unique tongue morphologies. Using phylogenetic generalized least square regressions, we found that tongue length (not size corrected) and the proportion of tongue that is bristled were both positively correlated to degree of nectarivory, while tongue length (relative to bill length), tongue depth (relative to bill depth) and tongue width (relative to bill width) were not correlated to nectarivory. Finally, we found no correlation between hyoid length (relative to bill length) and nectarivory, suggesting that the capacity for further tongue protrusion is unrelated to nectar dependence in honeyeaters. Similar studies should be conducted across other groups of avian nectarivores to expand our understanding of dietary ecomorphology beyond bill shape, which has been the focus of the majority of research on food handling adaptations in birds thus far.

## Introduction

Birds use the cranial apparatus as a tool to accomplish many tasks necessary for survival, and efficiently procuring and consuming food is one those critical tasks. The avian bill is often heralded as a classic example of morphological adaptation [e.g., 1,2], because of the intimate links between bill shape, diet, and functional performance in feeding processes [3–7]. Although diet is one factor that shapes bill evolution, there are also many intrinsic (e.g., common ancestry, developmental constraints, thermoregulation [8–16], vocalization and mechanosensation [17]) and extrinsic (e.g., intrasexual combat [18,19]) factors that can be important. There is a substantial body of literature that both qualitatively describes and quantitatively tests the evolutionary relationship between bill shape and ecological factors such as diet, but additional components of the avian feeding apparatus have received far less attention in this regard. The hyolingual apparatus (tongue and hyoid bones) is a vital feeding tool for many birds, as it is crucial for manipulating food in the mouth and moving it backwards towards the esophagus [20–22]. Moreover, in some lineages, the tongue and hyoid apparatus work with the bill to capture food (e.g., nectar feeders, filter feeders) [20–22]. Despite the importance of these structures during feeding, we know relatively little about how diet shapes the morphological evolution of the avian tongue and hyoid, as indicated by a relative dearth of quantitative macroevolutionary studies of hyolingual apparatus morphology.

There are a number of logistical reasons why there are far fewer studies on avian tongue and hyoid morphology than bill morphology. The biggest barrier is the limited availability of tongue and hyoid specimens in museum collections. Many studies of bill morphology make extensive use of birds prepared for collections. During the preparation of round skins, the most common specimen type in ornithological collections, the tongues and hyoids are typically discarded while the bill remains attached to the skin. In the case of intact bird specimens preserved in ethanol, the bill is often shut making the tongue inaccessible without using expensive and labor-intensive processes like computer tomography (CT) scanning, or through destructive sampling methods like dissection. If a CT scan is logistically possible, one is then presented with the challenge of how to successfully scan structures with little mineralization and therefore low X-ray contrast, rendering the tongue tissues nearly impossible to visualize. Avian tongues are typically keratinous, with some lineages such as waterfowl, penguins, and parrots having muscular tongues [20–22] – both of these tissue types often require electron-dense staining to be visible in CT scans [23–27]. If one has access to hyolingual apparatus specimens, either as part of an intact whole-body specimen or intentionally removed and stored in ethanol, the second major barrier is determining what measurements best quantify the shape of the structures. Three-dimensional geometric morphometrics (3D GMM) are a common, comprehensive technique for shape quantification [28,29], especially in macroevolutionary studies of morphology. However, using methods like 3D GMM is more difficult with soft tissue than bone. Soft tissue often lacks obvious homologous landmarks and can deform due to movement and/or preservation artifacts, which introduces noise to the shape analyses [30,31, but see 32]. This is especially true for tongues and hyoids because they are often flexible, smooth, and do not have external features that are clearly homologous between specimens.

To better understand the extent to which the avian tongue and hyoid vary in relation to differences in dietary ecology, it would be ideal to study a lineage of birds that subsist on a diet which requires integral use of the tongue and hyoid while feeding, and in which that diet is not present in sister taxa. Avian nectarivores are therefore a useful study system, as there are over 20 independent evolutions of specialized nectarivory in birds and nectar uptake involves extensive use of the tongue and hyoid [33]. Tongue and hyoid morphology are expected to influence drinking mechanics and nectar intake rates in avian nectarivores, which determines feeding efficiency and ultimately energy gained when foraging [34,35]. Thus, there is likely to be strong selection on the morphology of these structures in these lineages. Ample descriptive work has shown that all lineages of avian nectarivores have not converged on the same tongue morphology [literature summarized in 33], but the lack of quantitative morphological information has limited our ability to investigate macroevolutionary patterns of morphological evolution as it relates to nectarivory using phylogenetic comparative methods.

Honeyeaters (Meliphagidae) are an ideal group to address this question, as they are the second-most speciose group of avian nectarivores and exhibit high variability in their reliance on nectar [4,36]. Additionally, the outgroups to honeyeaters (thornbills, fairy wrens, pardalotes) are largely insectivorous and a shift to nectarivory occurred at the base of the Meliphagidae [4]. Within the honeyeater family, the range of reliance on nectar is so diverse that some honeyeater species have over 60% of their diet composed of nectar, whereas other species have reverted to full insectivory [4,36]; this dietary diversity stands in sharp contrast to other clades of avian nectarivores, like hummingbirds (Trochilidae), in which all studied species exhibit >60% of the diet composed of nectar [37] and, correspondingly, the tongue morphology is highly conserved across the family [38]. Previous descriptions of honeyeater tongue morphology have been made [39–41], but they have been limited to fewer than 20 species out of the ~ 180 species found in the family [42] and largely present qualitative descriptions and drawings [but see 41]. To our knowledge, there have been no data published on honeyeater hyoid morphology.

This study aims to characterize tongue and hyoid morphology across the Meliphagidae and determine whether diversity in these traits is correlated with dietary reliance on nectar. Morphology was characterized in two ways. First, we collected linear morphometric data of the tongue and hyoid that are likely to be related to the functional processes of nectar uptake. Because honeyeaters primarily use fluid trapping to collect nectar [43], a method in which surface tension causes an aliquot of nectar to be trapped between bristles or lamellae at the tongue tip, we predicted that the degree of nectarivory would be positively correlated with a longer tongue (increased reach), a greater proportion of the tongue that is bristled (increased surface area), and a wider and deeper tongue (increased volume). All of these features should theoretically increase nectar feeding efficiency. Also, as longer hyoid horns are associated with greater tongue protrusion which would be beneficial for nectar feeding [20,33,34], we predicted a positive correlation between the degree of nectarivory and hyoid horn length. As a complement to our linear morphometric measurements, we qualitatively described the gross morphology and microscopic anatomy of the tongue; from these descriptions we created categorical morphological states that were used in ecomorphological analyses, with the goal of capturing nuanced differences in morphology that would not be revealed by linear morphometrics. We aim to broaden our understanding of how the avian feeding apparatus has been modified in transitioning to a nectar-based diet, with emphasis on the potential for the tongue and hyoid to evolve adaptively in response to dietary differences.

## Methods

### Tongue and hyoid morphometrics

We examined preserved tongue specimens of 58 meliphagid species and 13 outgroup (non-nectarivore) species (S1 Table) and took a series of morphometric measurements specified below; the number of specimens per species ranged from 1–10 (S1 Table). Only a subset of specimens had the hyoid intact, so 38 meliphagid species and 5 outgroup species were examined for hyoid morphology (S1 Table); the number of specimens per species ranged from 1–4 (S1 Table). Specimens were from the University of Washington Burke Museum, the Harvard University Museum of Comparative Zoology, the UC Berkeley Museum of Vertebrate Zoology, the American Museum of Natural History, the Smithsonian, the Western Australian Museum, and the Queensland Museum (S1 Table). Specimens were kept in collections either in 70% ethanol or dried and tied to the leg of a study skin. Ethanol specimens consisted of the tongue, and sometimes the tongue and hyoid, dissected out during initial preparation of a study skin and kept in ethanol since the initial dissection. To make use of the dried specimens, we rehydrated them using a modified version of the procedure in [44] (see full protocol in S1 File). After the rehydration procedure all specimens were kept in 70% ethanol for long term storage. Each specimen’s preservation method is listed in S1 Table. All specimens were kept in a small dish of ethanol during measuring and photographing to prevent desiccation and warping.

We measured the specimens either under a dissecting microscope using a ruler or in ImageJ using photos taken under the microscope with a scale bar. All tongues were measured for total length (from tongue tip to center of the tongue base), bristle length (from tongue tip to the beginning of the bristles or fringes), width (distance between outer tongue walls in dorsal view, taken at the midpoint of the total tongue length), and depth (distance between outer tongue walls in lateral view, taken half way up the tongue) (S1 Fig). The portion of the tongue that is bristled was calculated as bristle length/total length of the tongue (S1 Fig). Most specimens with the hyoid intact had connective tissue covering the hyoid, making the junction between the ceratobranchial and epibranchial difficult to determine [Figure 17.4 in 20 for anatomical reference]. The length of each hyoid horn was measured as the combined ceratobranchial and epibranchial length (from junction of ceratobranchial with basihyal to the tip of the epibranchial). This length was measured by holding one end of a thin piece of string to the basihyal-ceratobranchial junction and wrapping the string around the horn, keeping it flush with the ceratobranchial and epibranchial, until it reached the tip of the epibranchial; the string was then removed and laid flat against a ruler and measured in length. If both hyoid horns were intact, then both were measured and a mean value was taken. If only one horn was intact then that single measurement was used. We also took bill measurements from the study skins or whole-body alcohol specimens associated with each specimen, so we could calculate tongue/bill morphometric ratios to account for bill size. We measured exposed culmen length, and bill width and bill depth (including both the maxillary and mandibular rhamphothecae) taken at the point where the nares end proximally. For specimens where the round skin could not be measured (12 specimens of 9 species from Smithsonian, see S1 Table) we used species mean values from the AVONET database [45] for culmen length, bill width, and bill depth. Raw data used in analyses is in S2 File and S3 File.

### Qualitative morphological descriptions

After taking linear morphometric measurements, we conducted morphological descriptions to capture the nuanced variation that was observed but could not be captured in linear measurements. In these qualitative descriptions of gross morphology, we focused on the presence and extent of certain structures, such as distal grooves and bristles, as well as the overall structure of the tongue, such as the thickness of the keratin walls and by extension the perceived stiffness of the structure. We found that there were six distinct tongue types within honeyeaters that represent six visually distinct morphological states. We assigned each species to one of these states, using the terminology Type 1–Type 6.

We then examined the microscopic anatomy of different tongue types to determine if there were corresponding internal anatomical differences. We examined the microscopic tongue morphology of *Ptilotula penicillata* (MCZ Ornithology 364080) which has a Type 1 tongue and *Melithreptus lunatus* (UWBM 76699) which has a Type 2 tongue. We then compared these data with published information on the microscopic tongue morphology of *Acanthorhynchus tenuirostris* (Type 4) and *Phylidonyris novaehollandiae* (Type 5) from [41]. Because internal microstructure could only be examined for four species, two newly described in this study, these results could not be included in macroevolutionary analyses. As such, further details on the methods of microscopic analysis, the descriptive results, and figures are in S4 File. The 3D models and μCT scan stacks used in S4 File will be deposited on the UW Burke Museum Morphosource profile.

### Dietary data

To examine the relationship between tongue morphology and diet, we used species-level diet data showing the proportion of feeding bouts that are nectar-feeding (from 0–1) [36,37]. For a species that takes no nectar (i.e., is fully insectivorous), this value would be zero, whereas for a species that subsists only on nectar this value would be 1. For the majority of species, we used diet data from [36]. For outgroups and species not found in [36], we used comparable data from the EltonTraits database [37]. For the tongue dataset, 41 out of the 58 species (71%) had diet data in [36] (S1 Table), and for the hyoid dataset 27 out of the 38 species (71%) had diet data in [36] (S1 Table).

### Statistics

#### Ancestral state reconstruction of tongue types.

All statistical analyses were performed in R v 4.5.1 [46]. For all phylogenetic comparative analyses, we used the most recent honeyeater time-calibrated molecular phylogeny from [42] pruned using the drop.tip() function from the ape package [47] to remove species that were not found in our morphological dataset. We then ran an ancestral state reconstruction analysis using stochastic character mapping to determine what the most likely ancestral tongue shape was for the Meliphagidae, as well as to calculate the number of predicted state changes and time spent in each state across the phylogeny. We used fitMk() in the phytools package v. 1.5−1 [48] to determine whether an unordered equal rates (ER) or all rates different (ARD) model would be best for ancestral state reconstruction, as determined by AIC. Based on the difference in AIC between the ER and ARD models (see Results), we used an ER model for stochastic character mapping. We ran an ancestral state reconstruction using stochastic character mapping [49] in the make.simmap() function in the ape package [47] with an equal rates model and nsim = 1000.

#### Correlation between hyolingual morphology and diet.

We investigated whether morphometrics and tongue types were correlated with the proportion of nectar in the diet. For analyses with morphometric data, we corrected the linear tongue and hyoid values for bill size by using ratios of tongue and hyoid metrics to bill metrics. We use tongue length/bill length, tongue width/bill width, tongue depth/bill depth, and hyoid length/bill length. Bristle proportion is already a ratio and so was not size corrected. We calculated species average values for each morphological variable to have a single value for each tip in the phylogenetic tree.

We began by quantifying phylogenetic signal in each variable using Blomberg’s K [50]. To test for phylogenetic signal, we used the phylosig() function in the phytools package v. 1.5−1 [48] and set to nsim = 1000. Upon finding that all but one variable (tongue depth) had significant phylogenetic signal, we used phylogenetic ANOVA, phylogenetic PCA (pPCA) and phylogenetic generalized least squares regressions (PGLS) to test our predictions. For these analyses, outgroup taxa were not included due to concerns that they would distort trait space and obscure patterns within the ingroup. We used phylogenetic ANOVA to determine whether tongue types differed significantly in their associated dietary nectar proportion, as tongue types are discrete states taking one of six possible values within the Meliphagidae (see descriptions in Results). We ran the phylogenetic ANOVA using the aov.phylo() function from the geiger package [51] set to nsim = 1000.

To determine the major axes of morphological diversity in our bill-size corrected linear morphometrics, we used the phyl.pca() function in the phytools package v. 1.5−1 [48]. Data were centered and scaled prior to the pPCA and two pPCAs were run, one for the tongue morphology only data set (58 meliphagid species) and one for the smaller data set that also included hyoid measurements (38 meliphagid species). We plotted the first three principal components because they collectively explained roughly 30% of the variation for both the tongue only dataset and tongue+hyoid dataset.

After conducting the pPCA for visualization, we used PGLS regressions to examine whether there was a correlation between each bill-size corrected morphological variable and the proportion of nectar in the diet, using Pagel’s λ to account for phylogenetic signal [52]. We predicted that the degree of nectarivory would be positively correlated with a longer tongue, a greater proportion of the tongue being bristled, a wider and deeper tongue, and longer hyoid horns. We used the gls() function in the nlme v 3.1–162 package [53] to run a PGLS for each variable with the correlation structure set to corPagel() and estimating parameters using maximized log likelihood. A second set of PGLS analyses were also run with the raw morphological data (tongue length, tongue width, tongue depth, bristle length, hyoid length) to determine whether the trends hold when there is no correction for bill size.

## Results

### Gross tongue morphology

**Fig 1:**
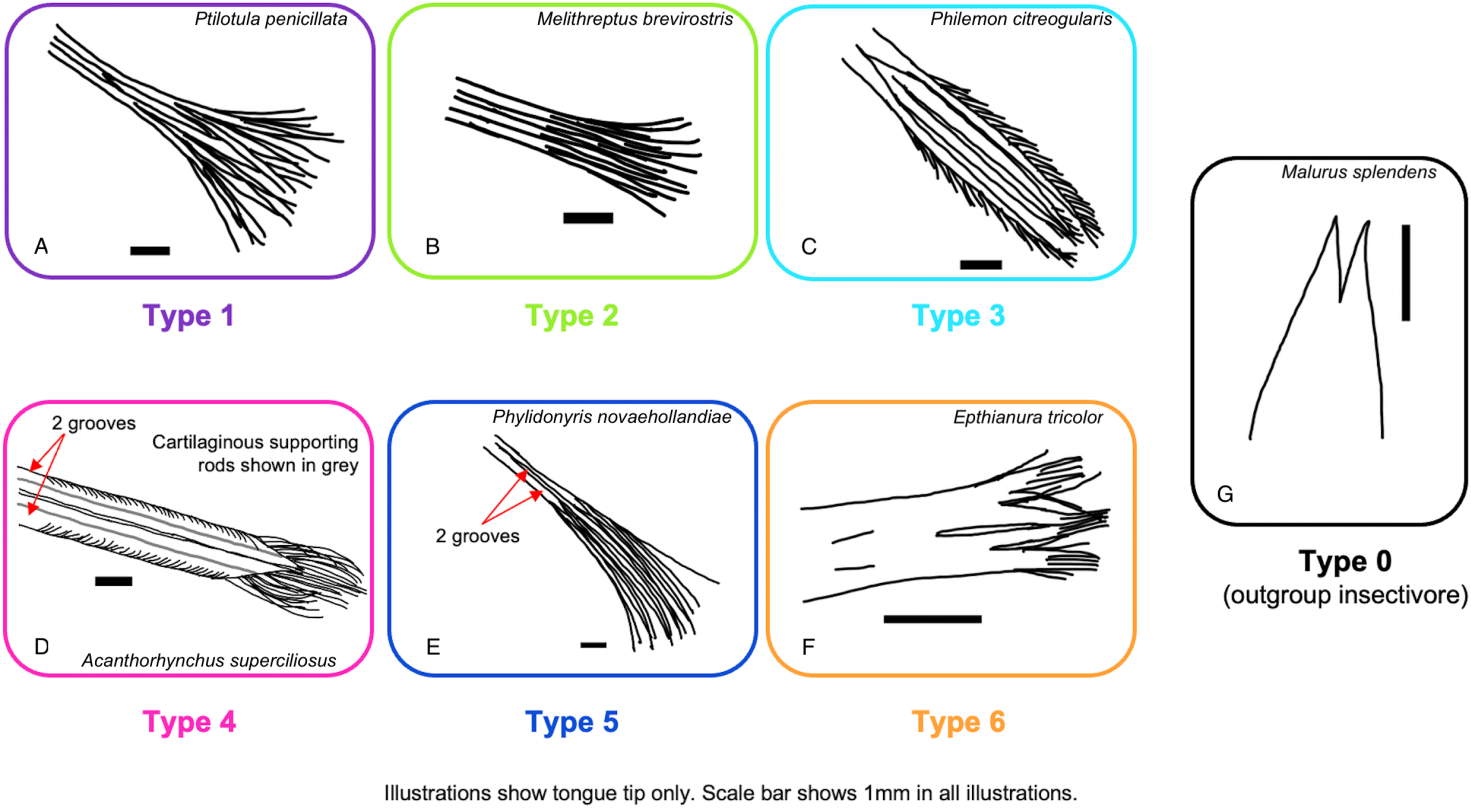
Schematic illustrations of six distinct tongue types found across the Meliphagidae in comparison to outgroup insectivores. Illustrations are drawn from the following specimens: Type 1: *Ptilotula penicillata* (UWBM 60839), Type 2: *Melithreptus brevirostris* (UWBM 76602), Type 3: *Philemon citreogularis* (UWBM 57671), Type 4: *Acanthorhynchus superciliosus* (UWBM 60869), Type 5: *Phylidonyris novaehollandiae* (USNM 612648), Type 6: *Epthianura tricolor* (WAM A13975), Type 0 (outgroup insectivore): *Malurus splendens* (WAM A5692). Illustrations were traced from photos using the Apple app Freeform. Photos of these and additional specimens demonstrating each tongue type are in S2 Fig.

**Fig 2:**
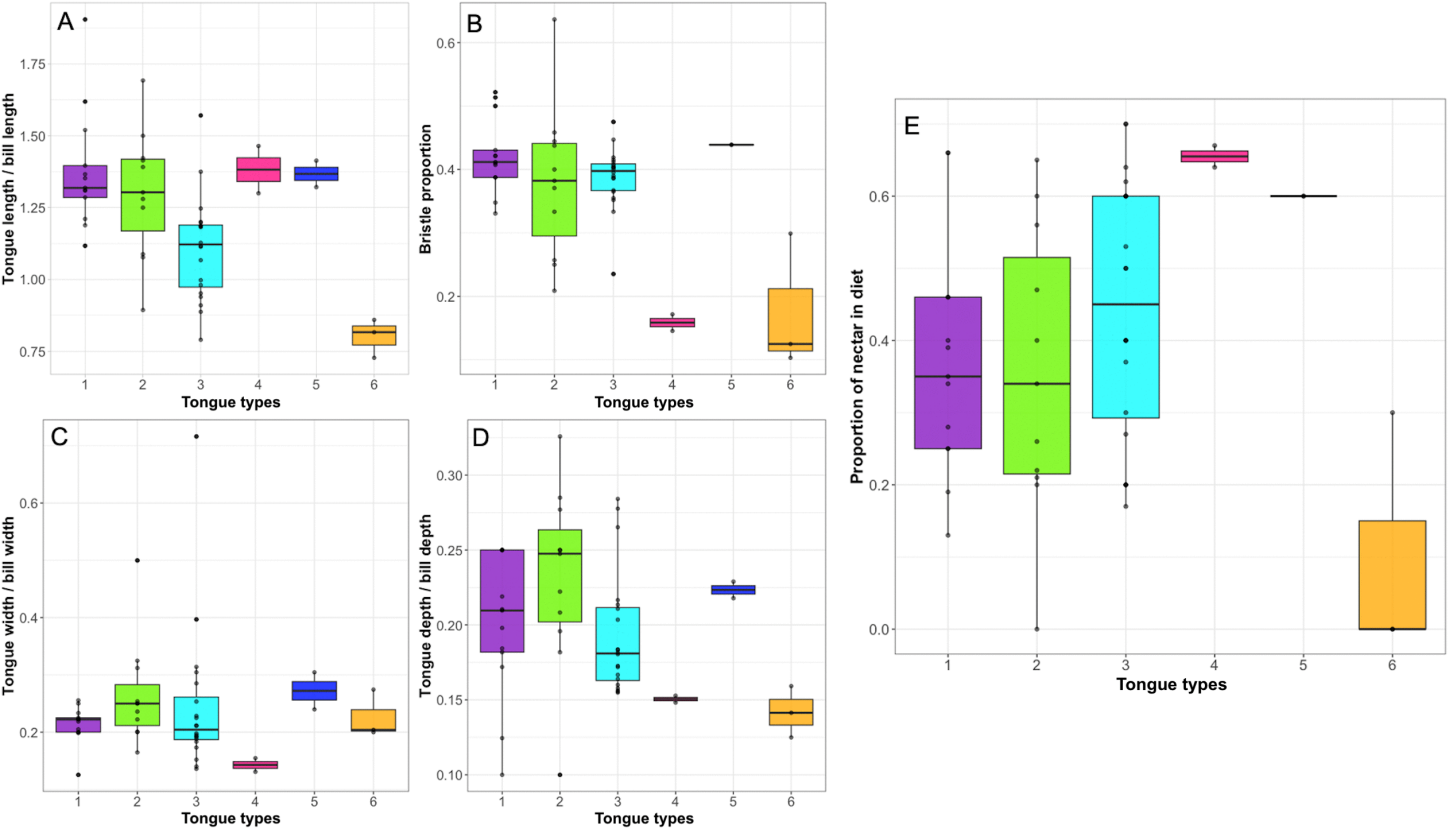
Differences in tongue morphometrics across tongue types found in the Meliphagidae and outgroup insectivores. A) Relative tongue length across tongue types, B) bristle proportion across tongue types, C) relative tongue width across tongue types, D) relative tongue depth across tongue types, E) proportion of dietary nectar across tongue types. Colors denote tongue types.

There is extensive diversity in the shape and structure of the distal portion of the tongue across the Meliphagidae (Fig 1 and S2 Fig). We found six tongue morphologies (tongue types), which were identifiable via clear, distinct features in the distal half of the tongue (Fig 1). The first tongue type is the classic “paintbrush” tongue as described by many previous publications (summarized in [33]). In this tongue type, the central trough of the tongue bifurcates at roughly half the tongue’s length and each branch bifurcates again to produce four distal grooves, each of which splits longitudinally many times to form many fine bristles (Fig 1A). The second tongue type is similar to the first, but the central bifurcation does not extend as far up the tongue body and the tongues are sturdier and stiffer (Fig 1B). The third tongue type departs from the paintbrush morphology in that there are minimal or no terminal bristles (Fig 1C). Type 3 tongues terminate in four grooves; the outer grooves have fringes (diagonal lacerations along the tissue) along their lateral margins (Fig 1C) and the medial grooves have few or no bristles at their tip. These tongues also vary in the number of fringes and relative size of the four grooves, with some species, specifically those of the genera *Plectorhyncha* and *Philemon* (S2 Fig), having four equally sized grooves and other species, such as those in the genera genera *Melilestes* and *Myzomela,* having two wider lateral grooves and two thinner medial grooves (S2 Fig). Tongue types 1–3 have similar tongue lengths relative to bill length (Fig 2A), bristle proportions (Fig 2B), tongue widths relative to bill width (Fig 2C), and tongue depths relative to bill depth (Fig 2D).

The fourth tongue type is only seen in members of the genus *Acanthorhynchus* (Fig 1D). These tongues are longer relative to bill length than Types 1–3 (Fig 2A), but their most distinctive features are the fact that they are composed of only two grooves, rather than the typical four, and there is a cartilaginous supporting rod along the ventral surface of each groove (S2 Fig); these features are illustrated in the histological images provided of *Acanthorhynchus superciliosus* in [41]. The distal end of the outer margin of each groove has many fringes (Fig 1D), but this does not extend far proximally. These tongues are narrower relative to bill width (Fig 2C) and shallower relative to bill depth (Fig 2D) than the other tongue types.

The fifth tongue type is only seen in members of the genus *Phylidonyris* (Fig 1E). Similar to the tongues of *Acanthorhynchus*, these tongues are longer relative to bill length than Types 1–3 (Fig 2A). In *Phylidonyris* the tongue looks similar to those in Type 1, but there are only two grooves and the bristles compose a larger portion of the tongue (Fig 1E and 2B). The presence of only two grooves in tongues of *Phylidonyris* is also shown in the histological images provided in [41] of *Phylidonyris novaehollandiae*. Interestingly, these tongues are wider relative to bill width (Fig 2C) and deeper relative to bill depth (Fig 2D) than most other tongue types, with Type 2 being most similar.

The sixth and final tongue type is seen only in the genus *Epthianura* (Fig 1F), one of the two genera that comprise the Australian chats, an insectivorous clade within the Meliphagidae. These tongues are shorter relative to bill length than the other tongue types seen across meliphagids, being more similar in length to insectivorous outgroups (Fig 2A), and have many small, short bristles on the distal tip (Fig 1F). These tongues also bifurcate roughly halfway along their length and they are similar in width to the other tongue types (except Type 4, Fig 2C) but shallower than in the other meliphagids examined, being more similar to insectivorous outgroups (Fig 2D). From the chats, we only found specimens of the genus *Epthianura* in collections, we could not locate any specimens of *Ashbyia*. Based on the drawings and comments of [54] for the genus *Ashbyia*, this morphology does not appear to be consistent between the two genera of chats. The tongue of *Ashbyia* is simpler with no grooves or bristles, being more similar to the tongue of an outgroup insectivore (Fig 1G).

### Ancestral state reconstruction of tongue types

**Fig 3:**
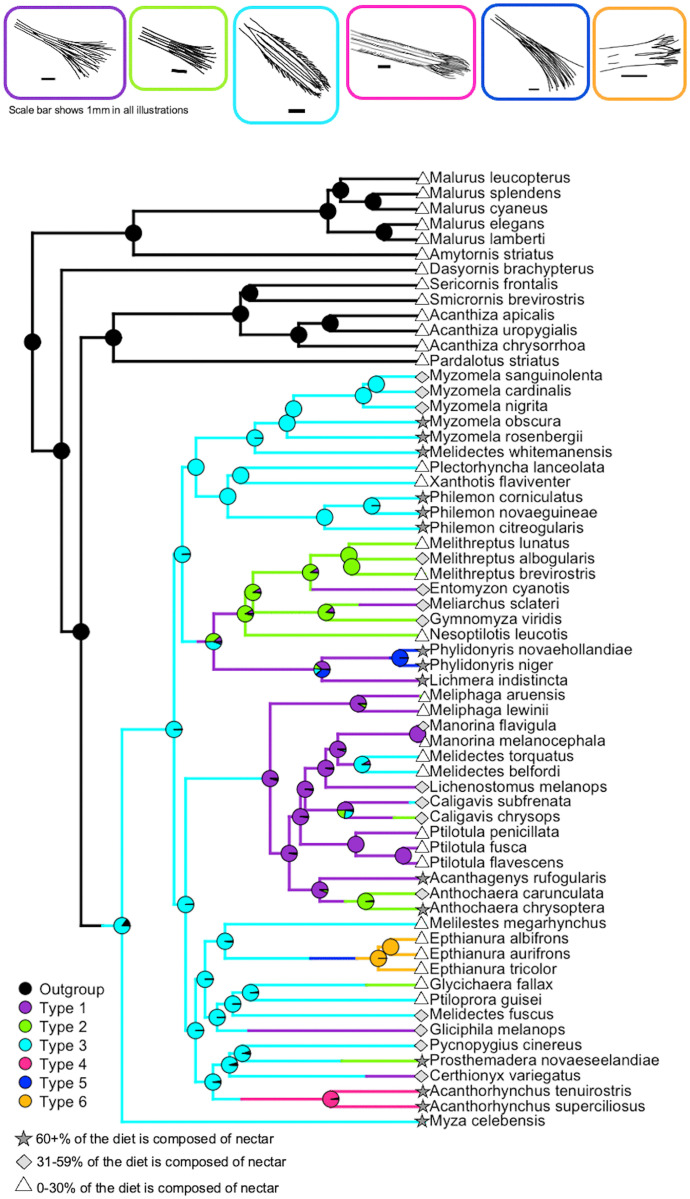
Ancestral state reconstruction of tongue type evolution in the Meliphagidae. Reconstruction determined via stochastic character mapping with equal rates model. Pies show the proportion of simulations in which a given state was selected via MCMC from a posterior distribution for a given node for this run. Dietary nectar consumption was split into three bins and marked using symbols. Dark star = 60 + % of the diet is nectar, light gray diamond = 31-59% of the diet is nectar, and white triangle = 0-30% of the diet is composed of nectar. Schematic illustrations of tongue types at the top of the figure are the same as those in Fig 1. Illustrations were traced from photos using the Apple app Freeform. Photos of these and additional specimens demonstrating each tongue type are in S2 Fig.

The ER model had an AIC of 159.51 and a log-likelihood −78.75, while ARD had an AIC of 195.21 and log-likelihood of −55.60. Given that the ΔAIC was > 2 between the two models in favor of the ER model, the ER model was used for stochastic character mapping. Stochastic character mapping trees had 22.17 changes between states on average. Within the Meliphagidae, the most common state was a Type 3 tongue, followed by Types 1 and 2 in roughly equal proportions (Table 1 and Fig 3). From the outgroup insectivorous type (Type 0) the most likely transition was to Type 3 as the ancestral state for the Meliphagidae (Fig 3). Across the meliphagids the most common change was to Type 1 (Table 1 and Fig 3), and Types 1–3 show higher rates of change than Types 4–6 (Table 1). Type 1 tongues are estimated to have evolved six times (Fig 3), whereas Type 2 tongues are estimated to have evolved five times (Fig 3). Type 3 tongues are estimated to have evolved three times throughout the tree, at the base of the Meliphagidae from an insectivorous ancestor and in the genus *Melidectes* and in *Caligavis subfrenata* (Fig 3). Types 4–6 are each estimated to have evolved once, as they are restricted to specific monophyletic genera (Fig 3).

**Table 1:** Results of ancestral state reconstruction of tongue types across Meliphagidae and outgroups.

Tongue type (state)	Proportion of time spent in state	Most common state change	
		To	Rate
0	0.28	3	1.14
1	0.14	2	2.87
2	0.13	1	2.14
3	0.38	1	3.11
4	0.028	1	0.17
5	0.019	1	0.57
6	0.024	1	0.19

Ancestral state reconstruction was run using stochastic character mapping with an equal rates model of state change.

### Morphological correlations with diet

#### Tongue types.

There was a significant phylogenetic signal in tongue types (S2 Table), and there was a significant difference in dietary nectar consumption across tongue types (*p* = 0.0085) (S3 Table and Fig 2E). Type 6 (insectivorous meliphagids) was associated with the lowest proportion of nectar in the diet (intercept_6_ = 0.10). Types 1–3 were associated with midrange nectar consumption (intercept_1_ = 0.37, intercept_2_ = 0.36, intercept_3_ = 0.45) and Types 4 and 5 (*Acanthorhynchus* and *Phylidonyris*, respectively) were associated with the highest proportion of nectar in the diet (intercept_4_ = 0.66, intercept_5_ = 0.60).

#### Linear morphometrics.

**Fig 4:**
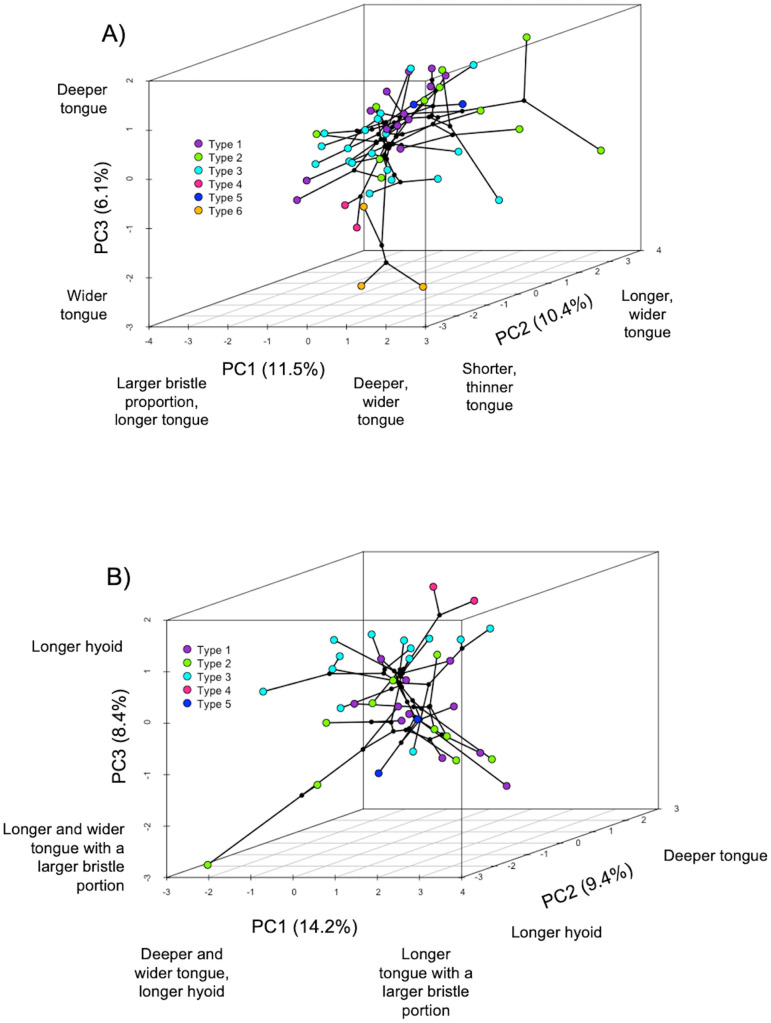
3D phylogenetic PCA of tongue and hyoid morphology. A) Tongue morphometrics only. B) Tongue and hyoid morphometrics. Labels under axes illustrate the loadings of each morphological variable. Colors indicate tongue types. See S4 Table for exact loadings.

All linear morphometric traits had phylogenetic signal except for tongue depth (S2 Table). In the pPCA for the tongue morphology dataset (58 meliphagid species, S1 Table), the first three principal components explain 28% of the variation (S4 Table and Fig 4A). In the pPCA for the tongue+hyoid dataset (38 meliphagid species, S1 Table) the first three principal components explain 32% of the variation (S4 Table and Fig 4B). In the tongue only pPCA, PC1 was determined almost equally by bristle proportion and relative tongue depth, though their loadings were in opposite directions, followed by relative tongue width and tongue length. PC2 was determined largely by relative tongue length and width, and PC3 was determined by relative tongue depth (S4 Table and Fig 4A). In the tongue+hyoid pPCA, PC1 was almost equally determined by relative tongue length, depth, and bristle proportion, whereas PC2 was determined by relative hyoid length and PC3 was determined by relative tongue width and length (S4 Table and Fig 4B).

**Fig 5:**
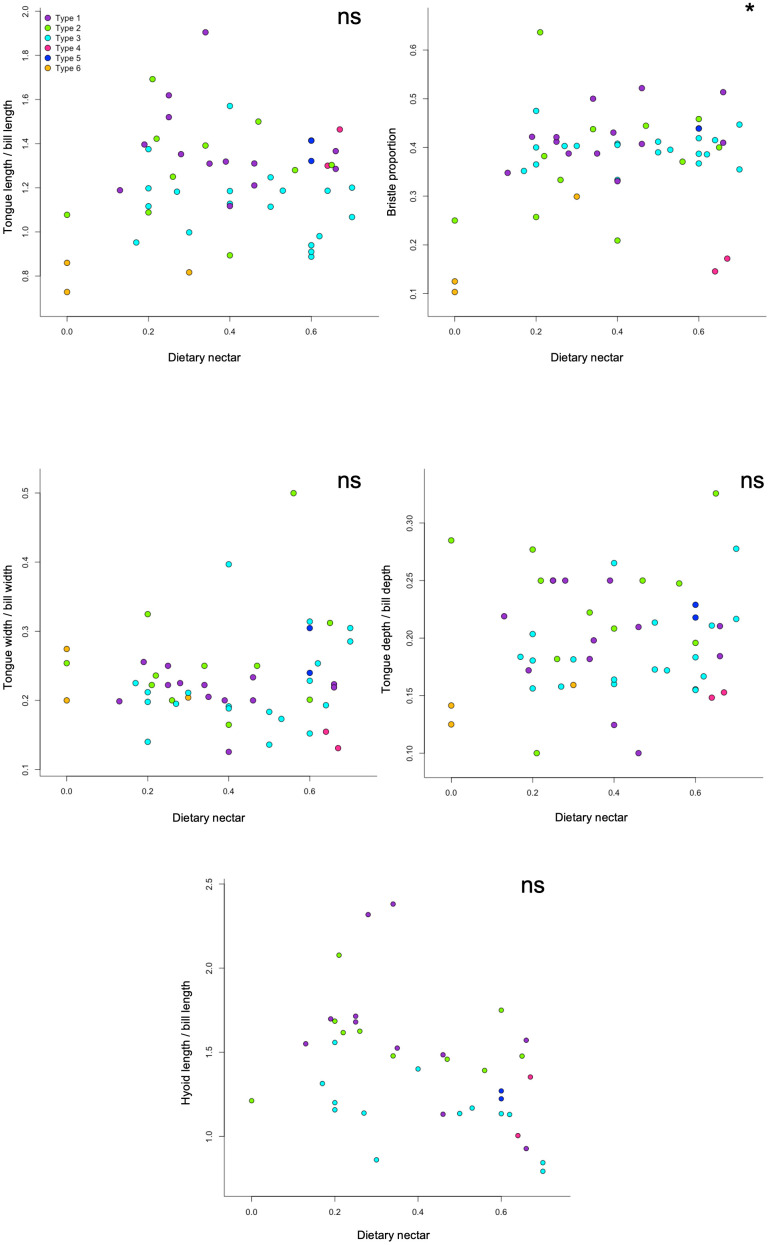
Correlations between dietary nectar percentage and tongue morphometrics. A) Relative tongue length, B) Bristle proportion, C) Relative tongue width, D) Relative tongue depth, and E) Relative hyoid length. Parameters for corresponding PGLS analyses are in Table 2. Colors correspond to tongue types. * indicates that the p-value from the PGLS regression was < 0.05, ns indicates the relationship was non-significant.

The proportion of the tongue that is bristled was significantly positively correlated to dietary nectar (Table 2 and Fig 5), whereas relative tongue length, width, and depth were not significantly correlated with nectarivory (Table 2 and Fig 5). There was a non-significant negative correlation between relative hyoid length and nectarivory (Table 2 and Fig 5). In the PGLS analyses of the raw data (i.e., tongue length, tongue width, tongue depth, and hyoid length not corrected for bill size), both tongue length and bristle proportion were significantly positively correlated with the degree of nectarivory, but all other variables were not significantly correlated with nectarivory (Table S5).

**Table 2:** Results of PGLS analyses for each bill –size-corrected morphological variable.

Variable	AIC	Log Likelihood	λ	Slope	Standard Error	t-value	*p*-value
Tongue length/ bill length	−16.95	12.47	0.76	0.18	0.15	1.14	0.26
Bristle proportion	−111.46	59.73	0.96	0.15	0.067	2.22	**0.031**
Tongue depth/ bill depth	−181.02	94.51	0.93	0.022	0.036	0.62	0.54
Tongue width/ bill width	−140.73	74.36	0.70	0.023	0.050	0.46	0.65
Hyoid length/ bill length	29.56	−10.78	0.69	−0.47	0.29	−1.59	0.12

Bolded *p*-value indicates significance. Significance determined as *p* < 0.05.

## Discussion

### Functional consequences of diversity in honeyeater tongue morphology

The distal half of the honeyeater tongue shows extensive morphological diversity between species, which we have classified into tongue types (Fig 1), whereas the anatomy in the proximal half of the tongue seems to be conserved across species (S4 File). Honeyeater tongues are typically described as having four grooves that terminate in many fine bristles, being similar to a paintbrush [e.g., [40]]. Of the honeyeaters sampled here, there are species that follow this description (Type 1 and Type 2 tongues, Fig 3), but many species that do not. Based on our sample, the ancestral state of the Meliphagidae tongue is estimated to be a Type 3 shape (Fig 3), which is different from the classic ‘paintbrush shape’ of the Type 1 and Type 2 tongues (Fig 1A-1B) in that there are minimal or no terminal bristles (Fig 1C). Rather than terminating in many fine bristles and evoking the shape of a paintbrush, Type 3 tongues maintain four distinct grooves up to the tongue tip and the lateral grooves have fringes along their lateral margins (Fig 1C). Interestingly, ancestral state reconstructions did find that the most common shape transition was to a Type 1 tongue (Table 1). We also found that Types 4–6 appear to be specific to the genera *Acanthorhynchus, Phylidonyris,* and *Epthianura*, respectively (Fig 3).

The diversity in honeyeater tongue morphology along with the fact that the degree of nectarivory differs significantly across tongue types is intriguing (S3 Table and Fig 2E) and raises the question of whether these differences in tongue shape have functional meaning that affects the process of nectar feeding. The only study to-date that has investigated the biomechanics of honeyeater feeding included five species: *Phylidonyris novaehollandiae*, *Manorina flavigula*, *Acanthagenys rufogularis*, *Ptilotula penicillata*, and *Certhionyx variegatus* [43]. All five of these species rely primarily on fluid trapping to consume nectar, a process in which the fluid is caught passively between bristles due to surface tension [43]. Four of the species (*Phylidonyris novaehollandiae*, *Manorina flavigula*, *Acanthagenys rufogularis*, and *Ptilotula penicillata*) used some degree of expansive filling (first described in hummingbirds [55]) to load nectar into the grooved body of the tongue, while the fifth species, *Certhionyx variegatus*, used capillary filling [43]. The five species examined in this previous study vary in tongue type. *Phylidonyris novaehollandiae* has a Type 5 tongue, which is unique to the genus *Phylidonyris*, whereas the remaining species (*Manorina flavigula*, *Acanthagenys rufogularis*, *Ptilotula penicillata,* and *Certhionyx variegatus*) have Type 1 tongues. The fact that all of these species rely primarily on fluid trapping suggests that there are a variety of tongue shapes that can perform this function equally well, although quantification of their corresponding nectar extraction efficiency is needed. Many-to-one-mapping of tongue form to function [56] could explain the evolutionary lability in the honeyeater tongue shape seen across the tree (Fig 3), with 22.17 state changes between tongue types on average across stochastic character mapping runs.

If there is many-to-one-mapping of tongue form to function for the process of fluid trapping, then the most important factors when determining feeding efficiency in species that use this mechanism may be the overall size of the tongue and the nectar properties of the flower the bird is feeding on [41,57]. Importantly, there was nothing notably unique about the tongue of *Certhionyx variegatus* compared to the tongues of the other three Type 1 species examined in [43] that would facilitate capillary filling versus expansive filling. It is possible that the distinction between those two mechanisms comes from emergent manipulation or deformation of the tongue during the feeding process due to interactions between the tongue and the bill.

### Convergence with other avian nectarivores

Of the tongue types we identified, the tongues of the genus *Acanthorhynchus* are remarkably convergent with the tongues of hummingbirds and sunbirds; compare with images from [24] and [58], respectively. This is not a surprising finding as *Acanthorhynchus* is the most nectarivorous genus within the Meliphagidae (Fig 2E). Both hummingbird tongues and *Acanthorhynchus* tongues have two grooves, cartilaginous supporting rods along the entire length of the tongue, and lateral fringes (Fig 1D and S2 Fig). The tongues of *Acanthorhynchus* have few bristles at the tongue tip (Fig 1D), making it unlikely that they could use those bristles for substantial fluid trapping. Although the feeding biomechanics of *Acanthorhynchus* have not been studied, we hypothesize that they might use expansive filling, like hummingbirds [55], or pressure-driven suction, like sunbirds [58], as their primary feeding mechanism instead of fluid trapping. If correct, this would constitute a high degree of morphological and functional convergence.

Previous work alludes to *Acanthorhynchus* likely using a different feeding mechanism than other honeyeaters. When presented with a series of nectar concentrations (10–60% wt/wt) at varying volumes (5,10, 50 microliters), *Anthochaera carunculata* and *Acanthagenys rufogularis* had different responses in their sugar intake rate compared to *Acanthorhynchus tenuirostris* [57]. For all species, the sugar intake rate was highest at 50 microliters, but the difference between the 50 microliter and 5 and 10 microliter treatments was much larger for *Anthochaera carunculata* and *Acanthagenys rufogularis* than for *Acanthorhynchus tenuirostris* [Fig 2 in 57]. Also, the sugar intake rate peaked at 50% concentration for *Anthochaera carunculata* and *Acanthagenys rufogularis*, but 30% for *Acanthorhynchus tenuirostris* with a sharper decline thereafter [Fig 2 in 57]. These differences could be explained by the different tongue morphology of the three species, beyond differences in tongue size [40,57], and by extension the potential for them to differ in feeding mechanics. We found that *Anthochaera carunculata* and *Acanthagenys rufogularis* have tongues with a larger bristle proportion relative to those of *Acanthorhynchus tenuirostris*. As a result, *Anthochaera carunculata* and *Acanthagenys rufogularis* can likely capture a larger aliquot of fluid on each lick using fluid trapping than can *Acanthorhynchus tenuirostris*, permitting that the bristle portion of the tongue is largely submerged. When the volume is reduced to 10 or 5 microliters, the intake rate dramatically declines in *Anthochaera carunculata* and *Acanthagenys rufogularis,* likely due to a reduction in the submersion depth of the bristled portion of their tongue [Table 3 in 40]. *Acanthorhynchus tenuirostris*, on the other hand, does not have a tongue built for fluid trapping and therefore a nectar collection mechanism more similar to expansive filling [55] or pressure-driven suction [58], would result in smaller differences in intake rates across variation in volume presentation. The difference in peak intake rate could also be explained by tongue morphology and feeding mechanics. If *Acanthorhynchus tenuirostris* uses a mechanism like that of hummingbirds or sunbirds, it is likely to be more sensitive to nectar concentration (due to the exponential relationship between sugar concentration and viscosity) because it requires more flow of nectar within the tongue body; fluid trapping conversely is likely to be less sensitive to concentration because there is less fluid flow.

### Morphological correlates with nectarivory

When correlating linear morphometrics with dietary nectar consumption, we found that a higher degree of nectarivory was significantly correlated with a greater bristle proportion and a longer tongue (non-size corrected) (Table 2 and S3 Fig 5). These results matched our prediction regarding bristle proportion and make functional sense, as a tongue with a greater proportion bristled has a greater surface area to engage in fluid trapping, the main way honeyeaters collect nectar [43]. There is nuance to this, however. As discussed above, the genus *Acanthorhynchus* has minimal bristles on the tongue tip but is highly nectarivorous, perhaps using a different feeding mechanism from fluid trapping.

These results partially matched our prediction regarding tongue length, in that only the raw data demonstrated a correlation with nectarivory. We predicted that a longer tongue would be postively correlated with the proportion of nectar in the diet, possibly because it allows for greater access to a wider range of floral resources or deeper submersion into a nectar pool. Tongue length relative to bill length did not correlate with nectarivory, suggesting that more nectarivorous honeyeaters do not have tongues that occupy a larger intraoral space to maximize elongation potential or nectar collection capacity. The significant correlation between raw tongue length and nectarivory suggests that an underlying correlation between another trait, for example bill and/or body size, and nectarivory may be present. There was no correlation between tongue width or depth and degree of nectarivory (Table 2 and S3 Table). Although there was no correlation with nectarivory, it is possible that tongue width and depth are constrained by internal bill anatomy, as there is clearly a relationship between tongue width and bill width and between tongue depth and bill depth (Fig S3). Though statistically non-significant, there was a negative correlation between nectarivory and hyoid length relative to bill length (Table 2 and Fig 5). In light of this result, we hypothesize that the honeyeater feeding apparatus is not modified for more efficient nectar feeding via an increased ability to protrude the tongue further past the bill tips. Rather, our results suggest that the honeyeater feeding apparatus is modified for nectarivory in the tongue only. The distance from the bill tip to the nectar surface has been shown to limit the nectar feeding efficiency of honeyeaters (*Acanthorhynchus superciliosus*, *Phylidonyris novaehollandiae,* and *Lichmera indistincta*) more so than that of hummingbirds (*Campylopterus hemileucurus, Heliodoxa jacula, Lampornis calolaema,* and *Phaethornis guy*) in experiments with artificial flowers [41]. In an interspecific comparison across honeyeaters using experiments with real flowers, a greater bill tip to nectar distance did not equate to lower feeding rate [59]. In fact, the species with a bill larger than the flower visited, which resulted in a longer bill tip to nectar distance, was the species with the largest tongue and the fastest rate of nectar consumption (microliters/second) [59]. So, while the finding of no correlation between hyoid length (both absolute and relative) and nectarivory is counter to the general expectations for increase nectar-feeding efficiency (summarized in [33]), it does lend support to the hypothesis that differences in feeding efficiency across honeyeaters are likely due to tongue size and/or morphological adaptations (e.g., bristles, grooves).

### Future directions

More studies on drinking mechanics of honeyeaters, especially those focused on the honeyeaters with tongue types that have not been studied biomechanically (e.g., genus *Acanthorhynchus*), will help to better elucidate the connections between diversity in tongue morphology and variability in tongue function across honeyeaters. Fluid dynamics models need to be constructed to quantify the degree to which tongue size, tongue morphology, such as bristle number and length, and nectar properties, like volume and viscosity, affect the volume of fluid captured per lick during the process of fluid trapping.

The robustness of our conclusions would greatly benefit from similar studies of nectarivores outside of the Meliphagidae. There are many qualitative descriptions of tongue morphology, and some on hyoid morphology, across other lineages of avian nectarivores, but quantitative morphological assessments are needed to determine if nectarivory results in morphological convergence, or similar vectors of morphological evolution, across avian nectarivores [34,43]. Given the degree of lability in tongue morphology found across honeyeaters, it would be interesting to investigate whether the hyolingual apparatus and its components evolve as their own evolutionary module in birds, similar to what has been demonstrated for various components of the cranium [60].

## Supporting information

S1 Fig Morphological measurements. Linear morphometrics measured on all tongues.
(PDF)

S2 Fig Photos of tongue types found across the Meliphagidae in comparison to outgroup insectivores. A) Type 1 tongues (*Manorina flavigula* (UWBM 57667) and *Ptilotula penicillata* (UWBM 60839)), B) Type 2 tongues (*Melithreptus brevirostris* (UWBM 76602) and *Melithreptus lunatus* (UWBM 76699)), C) Type 3 tongues (*Philemon citreogularis* (UWBM 57671) and *Melilestes megarhynchus* (UWBM 67917)), D) Type 4 tongues (*Acanthorhynchus tenuirostris* (UWBM 76471) and *Acanthorhynchus superciliosus* (UWBM 60869)), E) Type 5 tongues (*Phylidonyris novaehollandiae* (USNM 612648) and *Phylidonyris niger* (QM O.33431)), F) Type 6 tongues (*Epthianura tricolor* (WAM A13975) and *Epthianura aurifrons* (WAM A17143)), G) Type 0 tongues (outgroup insectivores, *Malurus splendens* (WAM A5692) and *Acanthiza apicalis* (WAM A17535)). Arrows in panel D illustrate the distinctive features of having two grooves, cartilaginous supporting rods that extend to the tip of the tongue, and lateral fringes on the distal portion of the tongue. Arrows in panel E illustrate the distinctive feature of having two grooves. Rulers in each image indicate millimeters. All photos were taken by A.E. Hewes.
(TIFF)

S3 Fig Relationships between corresponding tongue and bill morphometrics. A) Tongue length plotted against bill length, B) Tongue width plotted against bill width, C) Tongue depth plotted against bill depth, and D) Bristle proportion plotted against tongue length. Colors indicate tongue types.
(PNG)

S1 Table List of tongue only and tongue+hyoid specimens. Complete list of tongue only and tongue+hyoid specimens measured for this study. Museum abbreviations are as follows: UWBM = University of Washington Burke Museum, MCZ = Harvard Museum of Comparative Zoology, AMNH = American Museum of Natural History, USNM = Smithsonian, QM = Queensland Museum, WAM = Western Australian Museum, MVZ = UC Berkeley Museum of Vertebrate Zoology. Specimen number is listed in the nomenclature of the museum in which the specimen is housed. Diet source data abbreviations indicate whether data is from Miller et al. (2017) [EM] or Wilman et al. (2014) [ET]. Inclusion in the tongue only dataset is indicted with a Y, while a blank cell indicates that that specimen was not included in the dataset. Inclusion in the tongue+hyoid dataset is indicted with a Y, while a blank cell indicates that that specimen was not included in the dataset. Specimens may be included in the tongue only dataset but not the tongue+hyoid dataset if they did not have an intact hyoid or were part of a whole-body specimen such that the hyoid was embedded in muscle under the skin. Specimens may be included in the tongue+hyoid data but not the tongue only dataset if the hyoid was intact and could be measured, but the tongue itself was broken or damaged such that it was not appropriate to include it in the study.
(PDF)

S2 Table Results of Blomberg’s K test for phylogenetic signal. All traits except relative tongue depth show significant phylogenetic signal. Bolded p-values are significant. Significance determined as *p* < 0.05.
(PDF)

S3 Table Results of phylogenetic ANOVA of dietary nectar ~ tongue type. There is a significant correlation between dietary nectar consumption and tongue type. Bolded *p*-value indicates significance. Significance determined as p < 0.05.
(PDF)

S4 Table Results of pPCA for tongue only and tongue + hyoid morphological datasets. Values in rows show the loadings of each morphological variable in the pPCA. Percentages listed under each principal component show the percent variance explained by that component.
(PDF)

S5 Table Results of PGLS analyses for each raw morphological variable. Bolded p-value indicates significance. Significance determined as p < 0.05.
(PDF)

S1 File Tongue rehydration procedure. Steps modified from: Singer RA. Are dehydrated specimens a lost cause? A case study to reclaim dehydrated fluid-preserved specimens. Collection Forum. 2014;28(1–2): 16–20. All photos taken by A.E.Hewes.
(PDF)

S2 File Tongue morphometrics. Source data for analysis of tongue morphology (linear morphometrics) and tongue types.
(XLSX)

S3 File Hyoid morphometrics. Source data for analysis of hyoid morphology.
(XLSX)

S4 File Methods of microscopic anatomical analysis of *Melithreptus lunatus* and *Ptilotula penicillata*, descriptive results of microscopic tongue morphology, and accompanying illustrative figures. S4 Fig (histological cross sections of *Melithreptus lunatus*) and S5 Fig (microCT scan slices of *Ptilotula penicillata*) and included and figure legends are provided in the document.
(DOCX)
