# Supplementary material for: Morphological diversity in the honeyeater hyolingual apparatus and its relationship with nectarivory
Source: PLoS One. 2025 Dec 4;20(12):e0338219. doi: 10.1371/journal.pone.0338219 (PMC12677526; doi:10.1371/journal.pone.0338219)

Tongue rehydration procedure

Step	Description	Duration
1	Place a rack at the bottom of a screw top glass jar (jar needs to be sealable) and place tongue(s) on the rack. Pour 200mL of warm thymol solution (10 thymol crystals dissolved in 200mL DI H_2O) into jar and immediately screw on the lid to trap the steam. The tongue(s) should be suspended above, not immersed in, the thymol solution and the container should be sealed.	Leave tongue(s) in sealed jar for 1 week
2	Pour out the thymol solution and remove the rack. Place the tongue(s) in the jar and add 200mL DI H_2O. The tongue(s) should be fully submerged in the water and the container should be sealed.	Leave tongue(s) submerged for 24 hours
3	Pour out the DI water and replace with 200mL of 10% ethanol. The tongue(s) should be fully submerged in the water and the container should be sealed.	Leave tongue(s) submerged for 24 hours
4	Pour out the 10% ethanol and replace with 200mL of 20% ethanol. The tongue(s) should be fully submerged in the water and the container should be sealed.	Leave tongue(s) submerged for 24 hours
5	Pour out the 20% ethanol and replace with 200mL of 30% ethanol. The tongue(s) should be fully submerged in the water and the container should be sealed.	Leave tongue(s) submerged for 24 hours
6	Pour out the 30% ethanol and replace with 200mL of 40% ethanol. The tongue(s) should be fully submerged in the water and the container should be sealed.	Leave tongue(s) submerged for 24 hours
7	Pour out the 40% ethanol and replace with 200mL of 50% ethanol. The tongue(s) should be fully submerged in the water and the container should be sealed.	Leave tongue(s) submerged for 24 hours
8	Pour out the 50% ethanol and replace with 200mL of 60% ethanol. The tongue(s) should be fully submerged in the water and the container should be sealed.	Leave tongue(s) submerged for 24 hours
9	Pour out the 60% ethanol and replace with 200mL of 70% ethanol. The tongue(s) should be fully submerged in the water and the container should be sealed.	Leave tongue(s) submerged for 24 hours
10	Move tongue(s) to vial of fresh 70% ethanol for permanent storage.	Leave tongue(s) submerged indefinitely

Tongues in steam bath – Left: *Philemon corniculatus* (UWBM 76697); Right: *Melithreptus lunatus* (UWBM 76699)

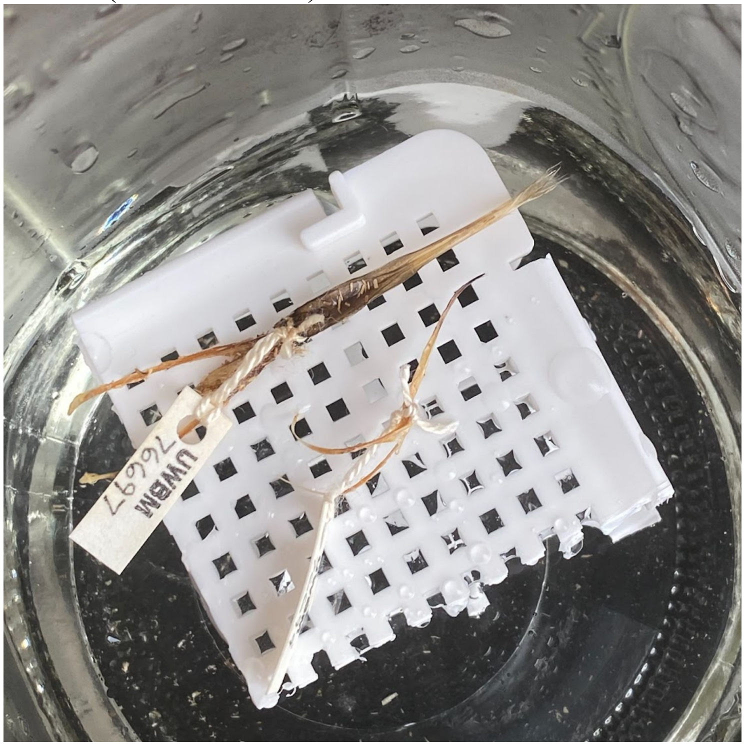

Philemon corniculatus (UWBM 76697) tongue throughout the rehydration process – A) fully desiccated, before rehydration; B) After steps 1-2, partially rehydrated; C) After steps 1-9, fully rehydrated in a dish of 70% ethanol

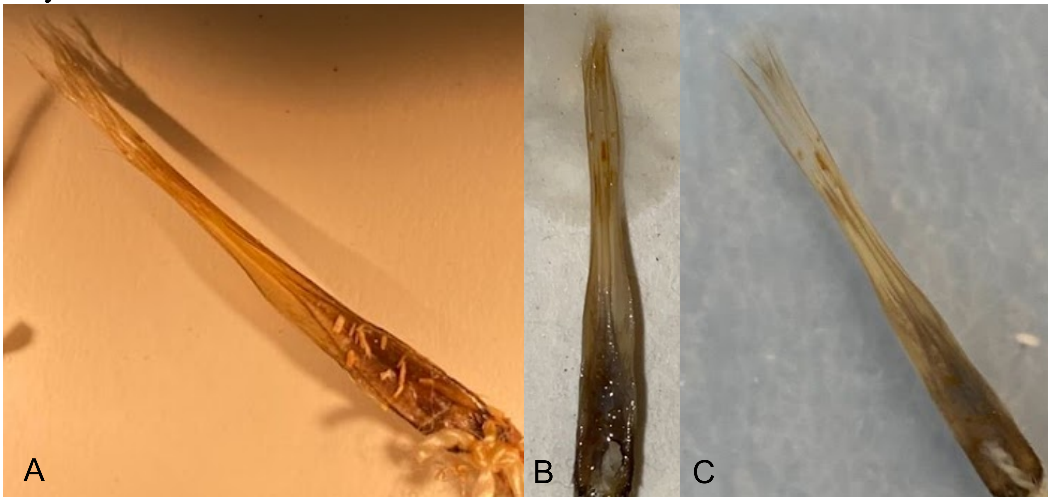

Supplement: S1 Fig — Linear morphometrics measured on all tongues. (PDF) [file pone.0338219.s001.pdf]
