# Supplementary figures and images for: Morphological diversity in the honeyeater hyolingual apparatus and its relationship with nectarivory
Source: PLoS One. 2025 Dec 4;20(12):e0338219. doi: 10.1371/journal.pone.0338219 (PMC12677526; doi:10.1371/journal.pone.0338219)

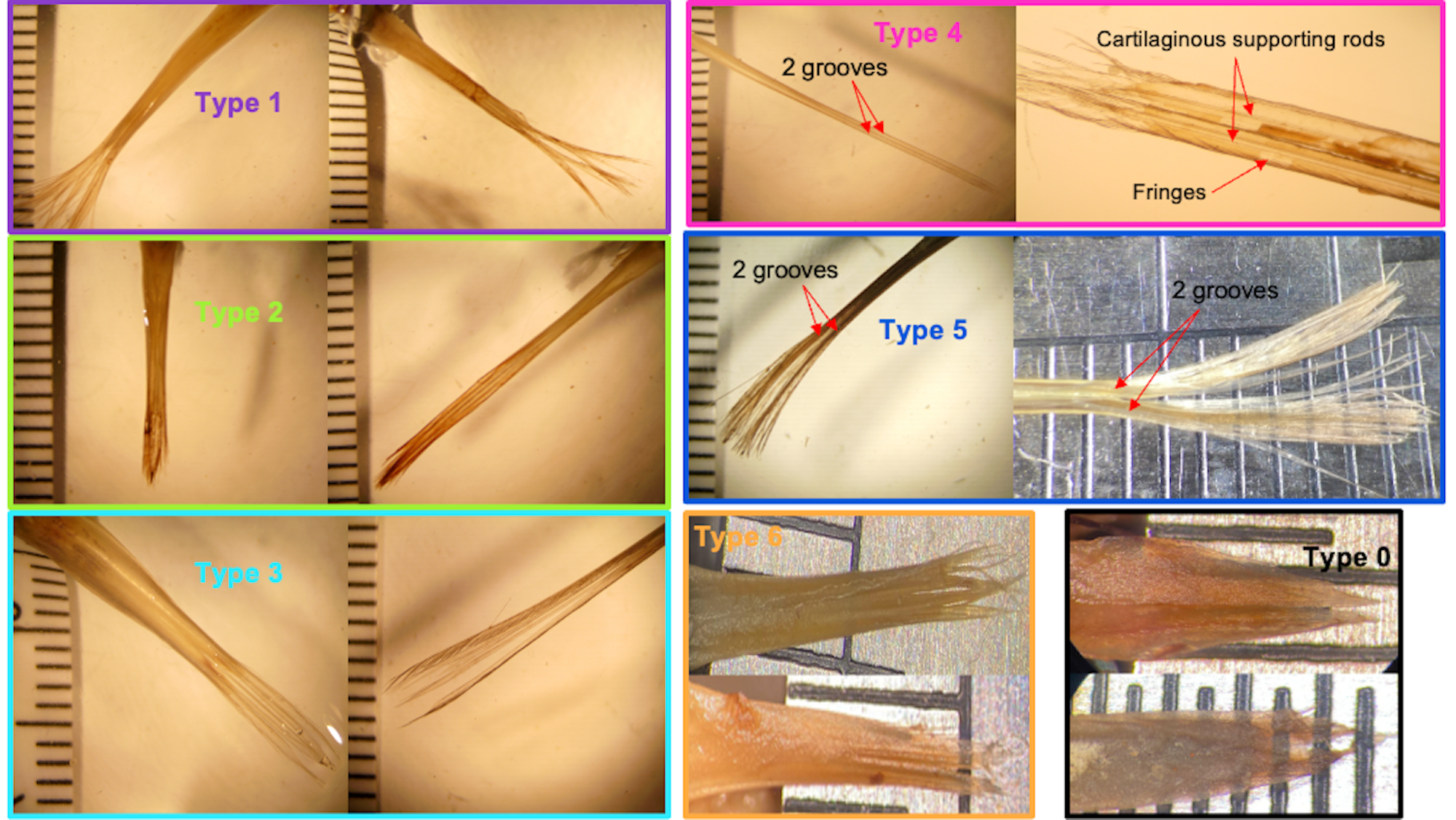

Supplement: S2 Fig — A) Type 1 tongues (Manorina flavigula (UWBM 57667) and Ptilotula penicillata (UWBM 60839)), B) Type 2 tongues (Melithreptus brevirostris (UWBM 76602) and Melithreptus lunatus (UWBM 76699)), C) Type 3 tongues (Philemon citreogularis (UWBM 57671) and Melilestes megarhynchus (UWBM 67917)), D) Type 4 tongues (Acanthorhynchus tenuirostris (UWBM 76471) and Acanthorhynchus superciliosus (UWBM 60869)), E) Type 5 tongues (Phylidonyris novaehollandiae (USNM 612648) and Phylidonyris niger (QM O.33431)), F) Type 6 tongues (Epthianura tricolor (WAM A13975) and Epthianura aurifrons (WAM A17143)), G) Type 0 tongues (outgroup insectivores, Malurus splendens (WAM A5692) and Acanthiza apicalis (WAM A17535)). Arrows in panel D illustrate the distinctive features of having two grooves, cartilaginous supporting rods that extend to the tip of the tongue, and lateral fringes on the distal portion of the tongue. Arrows in panel E illustrate the distinctive feature of having two grooves. Rulers in each image indicate millimeters. All photos were taken by A.E. Hewes. (TIFF) [file pone.0338219.s002.tiff]

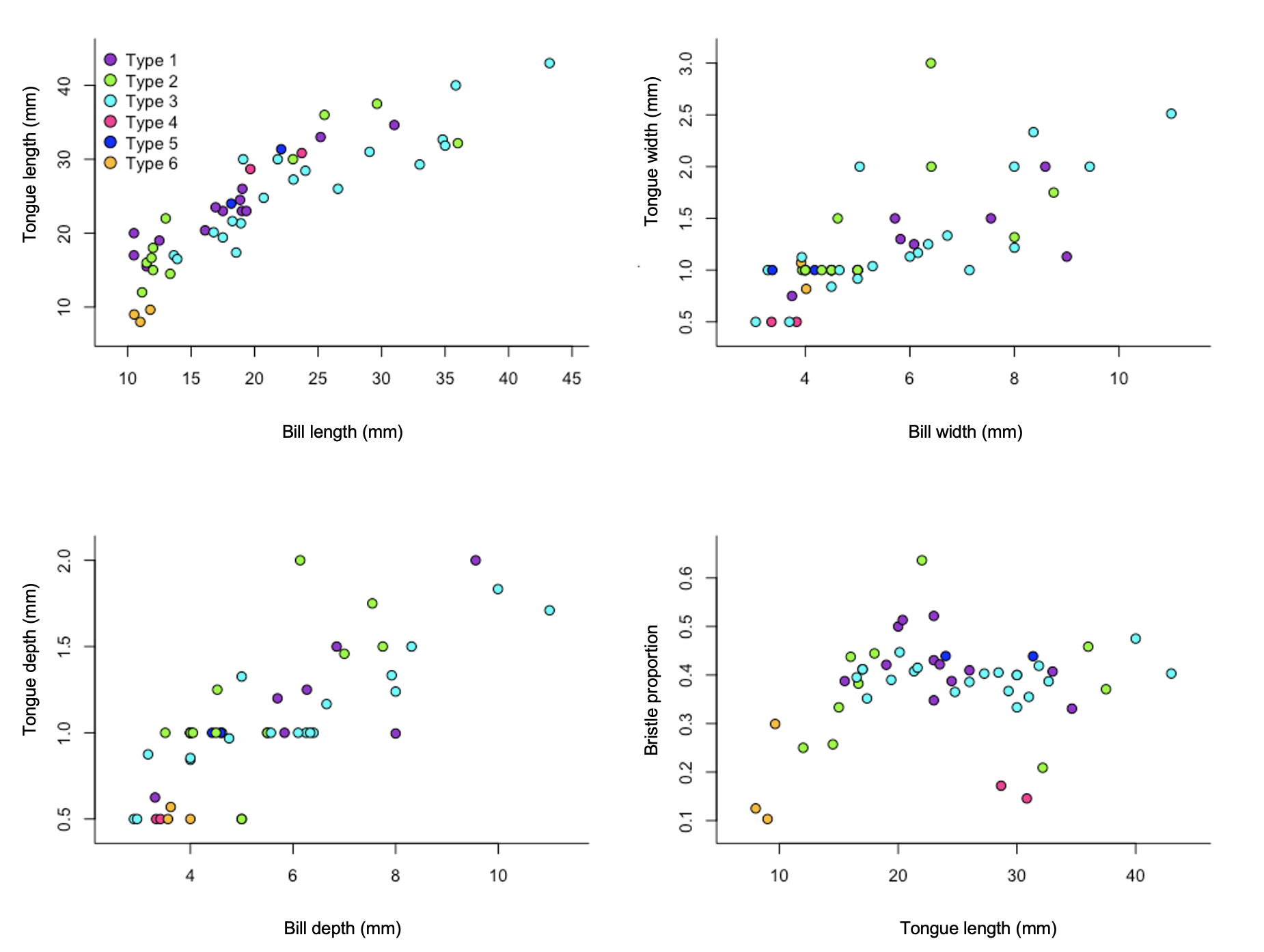

Supplement: S3 Fig — A) Tongue length plotted against bill length, B) Tongue width plotted against bill width, C) Tongue depth plotted against bill depth, and D) Bristle proportion plotted against tongue length. Colors indicate tongue types. (PNG) [file pone.0338219.s003.png]
