# Supplementary material for: Morphological diversity in the honeyeater hyolingual apparatus and its relationship with nectarivory
Source: PLoS One. 2025 Dec 4;20(12):e0338219. doi: 10.1371/journal.pone.0338219 (PMC12677526; doi:10.1371/journal.pone.0338219)
Supplement: S1 Table — Complete list of tongue only and tongue+hyoid specimens measured for this study. Museum abbreviations are as follows: UWBM = University of Washington Burke Museum, MCZ = Harvard Museum of Comparative Zoology, AMNH = American Museum of Natural History, USNM = Smithsonian, QM = Queensland Museum, WAM = Western Australian Museum, MVZ = UC Berkeley Museum of Vertebrate Zoology. Specimen number is listed in the nomenclature of the museum in which the specimen is housed. Diet source data abbreviations indicate whether data is from Miller et al. (2017) [EM] or Wilman et al. (2014) [ET]. Inclusion in the tongue only dataset is indicted with a Y, while a blank cell indicates that that specimen was not included in the dataset. Inclusion in the tongue+hyoid dataset is indicted with a Y, while a blank cell indicates that that specimen was not included in the dataset. Specimens may be included in the tongue only dataset but not the tongue+hyoid dataset if they did not have an intact hyoid or were part of a whole-body specimen such that the hyoid was embedded in muscle under the skin. Specimens may be included in the tongue+hyoid data but not the tongue only dataset if the hyoid was intact and could be measured, but the tongue itself was broken or damaged such that it was not appropriate to include it in the study. (PDF) [file pone.0338219.s004.pdf]

Museum	Specimen #	Species	Family	Preservation type	Diet data source	Tongue only dataset	Tongue +hyoid dataset
QM	O.32847	*Acanthagenys rufogularis*	Meliphagidae	Tongue in ethanol	EM	Y	Y
QM	O.33171	*Acanthagenys rufogularis*	Meliphagidae	Tongue in ethanol	EM	Y	Y
MCZ	23-034	*Acanthagenys rufogularis*	Meliphagidae	Rehydrated tongue	EM	Y	
MCZ	23-032	*Acanthagenys rufogularis*	Meliphagidae	Rehydrated tongue	EM	Y	
MCZ	23-027	*Acanthagenys rufogularis*	Meliphagidae	Rehydrated tongue	EM	Y	
WAM	A14638	*Acanthorhynchus superciliosus*	Meliphagidae	Whole specimen in ethanol	EM	Y	
WAM	A37449	*Acanthorhynchus superciliosus*	Meliphagidae	Whole specimen in ethanol	EM	Y	
UWBM	60869	*Acanthorhynchus superciliosus*	Meliphagidae	Rehydrated tongue	EM	Y	Y
MCZ	23-087	*Acanthorhynchus tenuirostris*	Meliphagidae	Rehydrated tongue	EM	Y	
MCZ	23-081	*Acanthorhynchus tenuirostris*	Meliphagidae	Rehydrated tongue	EM	Y	
MCZ	23-080	*Acanthorhynchus tenuirostris*	Meliphagidae	Rehydrated tongue	EM	Y	
MCZ	23-072	*Acanthorhynchus tenuirostris*	Meliphagidae	Rehydrated tongue	EM	Y	
USNM	612652	*Acanthorhynchus tenuirostris*	Meliphagidae	Tongue in ethanol	EM	Y	Y
UWBM	76471	*Acanthorhynchus tenuirostris*	Meliphagidae	Tongue in ethanol	EM	Y	Y
WAM	A34283	*Anthochaera carunculata*	Meliphagidae	Whole specimen in ethanol	EM	Y	
UWBM	57776	*Anthochaera carunculata*	Meliphagidae	Rehydrated tongue	EM		Y
USNM	612645	*Anthochaera carunculata*	Meliphagidae	Tongue in ethanol	EM	Y	Y
QM	O.33461	*Anthochaera chrysoptera*	Meliphagidae	Tongue in ethanol	EM	Y	Y
UWBM	76683	*Caligavis chrysops*	Meliphagidae	Rehydrated tongue	EM	Y	Y
MCZ	168100	*Caligavis subfrenata*	Meliphagidae	Rehydrated tongue	ET	Y	
WAM	A48670	*Certhionyx variegatus*	Meliphagidae	Whole specimen in ethanol	EM	Y	
WAM	A48859	*Certhionyx variegatus*	Meliphagidae	Whole specimen in ethanol	EM	Y	
WAM	A14302	*Conopophila rufogularis*	Meliphagidae	Whole specimen in ethanol	EM	Y	
QM	O.33363	*Entomyzon cyanotis*	Meliphagidae	Tongue in ethanol	EM		Y
QM	O.32926	*Entomyzon cyanotis*	Meliphagidae	Tongue in ethanol	EM	Y	Y
UWBM	76649	*Entomyzon cyanotis*	Meliphagidae	Rehydrated tongue	EM	Y	Y
WAM	A48860	*Epthianura albifrons*	Meliphagidae	Whole specimen in ethanol	EM	Y	
MCZ	SCO-125	*Epthianura albifrons*	Meliphagidae	Rehydrated tongue	EM	Y	
MCZ	SCO-124	*Epthianura albifrons*	Meliphagidae	Rehydrated tongue	EM	Y	
MCZ	SCO-123	*Epthianura albifrons*	Meliphagidae	Rehydrated tongue	EM	Y	
MCZ	SCO-122	*Epthianura albifrons*	Meliphagidae	Rehydrated tongue	EM	Y	
MCZ	SCO-121	*Epthianura albifrons*	Meliphagidae	Rehydrated tongue	EM	Y	
MCZ	SCO-120	*Epthianura albifrons*	Meliphagidae	Rehydrated tongue	EM	Y	
WAM	A17143	*Epthianura aurifrons*	Meliphagidae	Whole specimen in ethanol	EM	Y	
WAM	A13975	*Epthianura tricolor*	Meliphagidae	Whole specimen in ethanol	EM	Y	
QM	O.33352	*Epthianura tricolor*	Meliphagidae	Tongue in ethanol	EM	Y	
AMNH	1293	*Epthianura tricolor*	Meliphagidae	Whole specimen in ethanol	EM	Y	

WAM	A48669	*Gavicalis virescens*	Meliphagidae	Whole specimen in ethanol	EM	Y	
UWBM	60861	*Gliciphila melanops*	Meliphagidae	Rehydrated tongue	EM	Y	Y
MCZ	168130	*Glycichaera fallax*	Meliphagidae	Rehydrated tongue	EM	Y	Y
AMNH	5264	*Gymnomyza viridis*	Meliphagidae	Whole specimen in ethanol	ET	Y	
WAM	A48851	*Lichenostomus cratitius*	Meliphagidae	Whole specimen in ethanol	EM	Y	
UWBM	76551	*Lichenostomus melanops*	Meliphagidae	Rehydrated tongue	EM	Y	Y
WAM	A34154	*Lichmera indistincta*	Meliphagidae	Whole specimen in ethanol	EM	Y	
WAM	A48852	*Lichmera indistincta*	Meliphagidae	Whole specimen in ethanol	EM	Y	
WAM	A48853	*Lichmera indistincta*	Meliphagidae	Whole specimen in ethanol	EM	Y	
USNM	612736	*Lichmera indistincta*	Meliphagidae	Tongue in ethanol	EM	Y	Y
QM	O.33379	*Manorina flavigula*	Meliphagidae	Tongue in ethanol	EM	Y	Y
UWBM	57667	*Manorina flavigula*	Meliphagidae	Rehydrated tongue	EM	Y	Y
QM	O.33486	*Manorina melanocephala*	Meliphagidae	Tongue in ethanol	EM	Y	Y
AMNH	5268	*Meliarchus sclateri*	Meliphagidae	Whole specimen in ethanol	ET	Y	
MCZ	168119	*Melidectes belfordi*	Meliphagidae	Rehydrated tongue	ET	Y	Y
AMNH	5100	*Melidectes fuscus*	Meliphagidae	Whole specimen in ethanol	ET	Y	
MCZ	168168	*Melidectes torquatus*	Meliphagidae	Rehydrated tongue	ET	Y	Y
AMNH	563	*Melidectes whitemanensis*	Meliphagidae	Whole specimen in ethanol	ET	Y	
USNM	614972	*Melilestes megarhynchus*	Meliphagidae	Tongue in ethanol	ET	Y	Y
UWBM	67917	*Melilestes megarhynchus*	Meliphagidae	Rehydrated tongue	ET	Y	
MCZ	167949	*Melilestes megarhynchus*	Meliphagidae	Rehydrated tongue	ET	Y	Y
MCZ	168066	*Meliphaga aruensis*	Meliphagidae	Rehydrated tongue	ET	Y	Y
MCZ	168067	*Meliphaga aruensis*	Meliphagidae	Rehydrated tongue	ET	Y	Y
QM	O.33665	*Meliphaga lewinii*	Meliphagidae	Tongue in ethanol	EM	Y	Y
UWBM	76681	*Meliphaga lewinii*	Meliphagidae	Rehydrated tongue	EM	Y	Y
UWBM	76710	*Melithreptus albogularis*	Meliphagidae	Rehydrated tongue	EM	Y	Y
UWBM	76602	*Melithreptus brevirostris*	Meliphagidae	Rehydrated tongue	EM	Y	Y
WAM	A16588	*Melithreptus gularis*	Meliphagidae	Whole specimen in ethanol	EM	Y	
WAM	none	*Melithreptus gularis*	Meliphagidae	Whole specimen in ethanol	EM	Y	
QM	O.33644	*Melithreptus lunatus*	Meliphagidae	Tongue in ethanol	EM	Y	Y
MCZ	SCO-162	*Melithreptus lunatus*	Meliphagidae	Rehydrated tongue	EM	Y	
MCZ	23-086	*Melithreptus lunatus*	Meliphagidae	Rehydrated tongue	EM	Y	
MCZ	23-085	*Melithreptus lunatus*	Meliphagidae	Rehydrated tongue	EM	Y	
MCZ	23-084	*Melithreptus lunatus*	Meliphagidae	Rehydrated tongue	EM	Y	
MCZ	23-083	*Melithreptus lunatus*	Meliphagidae	Rehydrated tongue	EM	Y	
MCZ	23-082	*Melithreptus lunatus*	Meliphagidae	Rehydrated tongue	EM	Y	
MCZ	23-078	*Melithreptus lunatus*	Meliphagidae	Rehydrated tongue	EM	Y	
USNM	612646	*Melithreptus lunatus*	Meliphagidae	Tongue in ethanol	EM	Y	Y
UWBM	76699	*Melithreptus lunatus*	Meliphagidae	Rehydrated tongue	EM	Y	Y

MVZ	KMCR700	*Myza celebensis*	Meliphagidae	Tongue in ethanol	ET	Y	Y
AMNH	595	*Myzomela cardinalis*	Meliphagidae	Whole specimen in ethanol	ET	Y	
AMNH	5103	*Myzomela cardinalis*	Meliphagidae	Whole specimen in ethanol	ET	Y	
MCZ	167869	*Myzomela nigrita*	Meliphagidae	Rehydrated tongue	ET	Y	Y
MCZ	167870	*Myzomela nigrita*	Meliphagidae	Rehydrated tongue	ET	Y	Y
WAM	A26151	*Myzomela obscura*	Meliphagidae	Whole specimen in ethanol	EM	Y	
AMNH	5104	*Myzomela obscura*	Meliphagidae	Whole specimen in ethanol	EM	Y	
MCZ	167885	*Myzomela rosenbergii*	Meliphagidae	Rehydrated tongue	ET	Y	Y
MCZ	167882	*Myzomela rosenbergii*	Meliphagidae	Rehydrated tongue	ET	Y	
MCZ	167889	*Myzomela rosenbergii*	Meliphagidae	Rehydrated tongue	ET	Y	Y
MCZ	167887	*Myzomela rosenbergii*	Meliphagidae	Rehydrated tongue	ET	Y	Y
QM	O.33439	*Myzomela sanguinolenta*	Meliphagidae	Tongue in ethanol	EM	Y	Y
USNM	612651	*Myzomela sanguinolenta*	Meliphagidae	Tongue in ethanol	EM	Y	Y
UWBM	76729	*Nesoptilotis leucotis*	Meliphagidae	Rehydrated tongue	EM	Y	Y
QM	O.32797	*Philemon citreogularis*	Meliphagidae	Tongue in ethanol	EM	Y	Y
MCZ	SCO-134	*Philemon citreogularis*	Meliphagidae	Rehydrated tongue	EM	Y	
MCZ	SCO-130	*Philemon citreogularis*	Meliphagidae	Rehydrated tongue	EM	Y	
MCZ	23-055	*Philemon citreogularis*	Meliphagidae	Rehydrated tongue	EM	Y	
UWBM	57671	*Philemon citreogularis*	Meliphagidae	Rehydrated tongue	EM	Y	Y
QM	O.32772	*Philemon citreogularis*	Meliphagidae	Tongue in ethanol	EM	Y	Y
QM	O.33381	*Philemon corniculatus*	Meliphagidae	Tongue in ethanol	EM	Y	Y
USNM	612653	*Philemon corniculatus*	Meliphagidae	Tongue in ethanol	EM	Y	Y
UWBM	76697	*Philemon corniculatus*	Meliphagidae	Rehydrated tongue	EM	Y	Y
AMNH	5109	*Philemon novaeguineae*	Meliphagidae	Whole specimen in ethanol	ET	Y	
QM	O.33431	*Phylidonyris niger*	Meliphagidae	Tongue in ethanol	EM	Y	Y
QM	O.33432	*Phylidonyris niger*	Meliphagidae	Tongue in ethanol	EM	Y	
WAM	A27418	*Phylidonyris novaehollandiae*	Meliphagidae	Whole specimen in ethanol	EM	Y	
WAM	A14500	*Phylidonyris novaehollandiae*	Meliphagidae	Whole specimen in ethanol	EM	Y	
WAM	A23038	*Phylidonyris novaehollandiae*	Meliphagidae	Whole specimen in ethanol	EM	Y	
USNM	612647	*Phylidonyris novaehollandiae*	Meliphagidae	Tongue in ethanol	EM	Y	Y
USNM	612648	*Phylidonyris novaehollandiae*	Meliphagidae	Tongue in ethanol	EM	Y	Y
USNM	612649	*Phylidonyris novaehollandiae*	Meliphagidae	Tongue in ethanol	EM	Y	Y
USNM	612650	*Phylidonyris novaehollandiae*	Meliphagidae	Tongue in ethanol	EM	Y	Y
UWBM	57795	*Plectorhyncha lanceolata*	Meliphagidae	Rehydrated tongue	EM	Y	Y
MCZ	SCO-166	*Plectorhyncha lanceolata*	Meliphagidae	Rehydrated tongue	EM	Y	
MCZ	SCO-165	*Plectorhyncha lanceolata*	Meliphagidae	Rehydrated tongue	EM	Y	
MCZ	SCO-164	*Plectorhyncha lanceolata*	Meliphagidae	Rehydrated tongue	EM	Y	
MCZ	SCO-114	*Plectorhyncha lanceolata*	Meliphagidae	Rehydrated tongue	EM	Y	
MCZ	23-037	*Plectorhyncha lanceolata*	Meliphagidae	Rehydrated tongue	EM	Y	
MCZ	23-036	*Plectorhyncha lanceolata*	Meliphagidae	Rehydrated tongue	EM	Y	

MCZ	23-026	*Plectorhyncha lanceolata*	Meliphagidae	Rehydrated tongue	EM	Y	
AMNH	5121	*Prosthemadera novaeseelandiae*	Meliphagidae	Whole specimen in ethanol	ET	Y	
UWBM	79583	*Prosthemadera novaeseelandiae*	Meliphagidae	Rehydrated tongue	ET	Y	Y
MCZ	168074	*Ptiloprora guisei*	Meliphagidae	Rehydrated tongue	ET	Y	
MCZ	168072	*Ptiloprora guisei*	Meliphagidae	Rehydrated tongue	ET	Y	Y
AMNH	570	*Ptiloprora guisei*	Meliphagidae	Whole specimen in ethanol	ET	Y	
AMNH	646	*Ptiloprora guisei*	Meliphagidae	Whole specimen in ethanol	ET	Y	
WAM	A14300	*Ptilotula flavescens*	Meliphagidae	Whole specimen in ethanol	EM	Y	
UWBM	60915	*Ptilotula flavescens*	Meliphagidae	Rehydrated tongue	EM	Y	Y
UWBM	76519	*Ptilotula fusca*	Meliphagidae	Rehydrated tongue	EM	Y	Y
WAM	A27522	*Ptilotula keartlandi*	Meliphagidae	Whole specimen in ethanol	EM	Y	
UWBM	60839	*Ptilotula penicillata*	Meliphagidae	Rehydrated tongue	EM	Y	Y
WAM	A13968	*Ptilotula plumula*	Meliphagidae	Whole specimen in ethanol	EM	Y	
WAM	A13971	*Purnella albifrons*	Meliphagidae	Whole specimen in ethanol	EM	Y	
MCZ	168145	*Pycnopygius cinereus*	Meliphagidae	Rehydrated tongue	ET	Y	Y
MCZ	168147	*Pycnopygius cinereus*	Meliphagidae	Rehydrated tongue	ET	Y	Y
MCZ	168146	*Pycnopygius cinereus*	Meliphagidae	Rehydrated tongue	ET	Y	Y
WAM	A14305	*Stomiopera unicolor*	Meliphagidae	Whole specimen in ethanol	EM	Y	
WAM	A13987	*Stomiopera unicolor*	Meliphagidae	Whole specimen in ethanol	EM	Y	
MCZ	168180	*Xanthotis flaviventer*	Meliphagidae	Rehydrated tongue	EM	Y	Y
MCZ	168182	*Xanthotis flaviventer*	Meliphagidae	Rehydrated tongue	EM	Y	Y
WAM	A17764	*Acanthiza apicalis*	Acanthizidae	Whole specimen in ethanol	ET	Y	
WAM	A17535	*Acanthiza apicalis*	Acanthizidae	Whole specimen in ethanol	ET	Y	
WAM	A34359	*Acanthiza chrysorrhoa*	Acanthizidae	Whole specimen in ethanol	ET	Y	
WAM	A48666	*Acanthiza chrysorrhoa*	Acanthizidae	Whole specimen in ethanol	ET	Y	
WAM	A48861	*Acanthiza uropygialis*	Acanthizidae	Whole specimen in ethanol	ET	Y	
WAM	A23041	*Sericornis frontalis*	Acanthizidae	Whole specimen in ethanol	ET	Y	
WAM	A23029	*Sericornis frontalis*	Acanthizidae	Whole specimen in ethanol	ET	Y	
WAM	A23021	*Sericornis frontalis*	Acanthizidae	Whole specimen in ethanol	ET	Y	
WAM	A27542	*Smicrornis brevirostris*	Acanthizidae	Whole specimen in ethanol	ET	Y	
WAM	A38077	*Smicrornis brevirostris*	Acanthizidae	Whole specimen in ethanol	ET	Y	
WAM	A13989	*Smicrornis brevirostris*	Acanthizidae	Whole specimen in ethanol	ET	Y	
WAM	A27168	*Dasyornis brachypterus*	Dasyornithidae	Tongue in ethanol	ET	Y	Y
WAM	A18391	*Amytornis striatus*	Maluridae	Whole specimen in ethanol	ET	Y	Y
QM	O.33630	*Malurus cyaneus*	Maluridae	Tongue in ethanol	ET	Y	Y
WAM	A48660	*Malurus elegans*	Maluridae	Whole specimen in ethanol	ET	Y	

WAM	A14307	*Malurus lamberti*	Maluridae	Whole specimen in ethanol	ET	Y	
QM	O.32656	*Malurus lamberti*	Maluridae	Tongue in ethanol	ET	Y	Y
AMNH	4094	*Malurus lamberti*	Maluridae	Whole specimen in ethanol	ET	Y	
WAM	A13982	*Malurus leucopterus*	Maluridae	Whole specimen in ethanol	ET	Y	
WAM	A13983	*Malurus leucopterus*	Maluridae	Whole specimen in ethanol	ET	Y	
WAM	A5692	*Malurus splendens*	Maluridae	Whole specimen in ethanol	ET	Y	
WAM	A27498	*Pardalotus striatus*	Pardalotidae	Whole specimen in ethanol	ET	Y	
QM	O.33400	*Pardalotus striatus*	Pardalotidae	Tongue in ethanol	ET	Y	Y
