# Supplementary material for: Morphological diversity in the honeyeater hyolingual apparatus and its relationship with nectarivory
Source: PLoS One. 2025 Dec 4;20(12):e0338219. doi: 10.1371/journal.pone.0338219 (PMC12677526; doi:10.1371/journal.pone.0338219)
Supplement: S2 Table — All traits except relative tongue depth show significant phylogenetic signal. Bolded p-values are significant. Significance determined as p < 0.05. (PDF) [file pone.0338219.s005.pdf]

**Morphological Variable**	**Blomberg's K**	***p*-value**<br>(H_0: no phylogenetic signal)
Tongue type	2.151	**0.001**
Tongue length/bill length	1.473	**0.001**
Bristle proportion<br>(bristle length/tongue length)	1.949	**0.001**
Tongue depth/bill depth	0.371	0.092
Tongue width/bill width	0.767	**0.001**
Hyoid length/bill length	0.553	**0.033**
