# Supplementary material for: Morphological diversity in the honeyeater hyolingual apparatus and its relationship with nectarivory
Source: PLoS One. 2025 Dec 4;20(12):e0338219. doi: 10.1371/journal.pone.0338219 (PMC12677526; doi:10.1371/journal.pone.0338219)
Supplement: S3 Table — There is a significant correlation between dietary nectar consumption and tongue type. Bolded p-value indicates significance. Significance determined as p < 0.05. (PDF) [file pone.0338219.s006.pdf]

	**Df**	**Sums of Squares**	**Mean Square**	**F-value**	***p*-value**
Tongue type	5	0.56	0.11	3.6	**0.0085**
Residuals	44	1.4	0.031		
