# Supplementary material for: Morphological diversity in the honeyeater hyolingual apparatus and its relationship with nectarivory
Source: PLoS One. 2025 Dec 4;20(12):e0338219. doi: 10.1371/journal.pone.0338219 (PMC12677526; doi:10.1371/journal.pone.0338219)
Supplement: S4 Table — Values in rows show the loadings of each morphological variable in the pPCA. Percentages listed under each principal component show the percent variance explained by that component. (PDF) [file pone.0338219.s007.pdf]

**Morphological Variable (tongue only dataset)**	**PC1** 11.5%	**PC2** 10.4%	**PC3** 6.1%	**PC4** 4.03%
Tongue length/bill length	-0.47	0.76	0.036	-0.45
Bristle proportion	-0.68	0.34	0.43	0.49
Tongue depth/bill depth	0.69	0.17	0.69	-0.11
Tongue width/bill width	0.54	0.73	-0.33	0.26
**Morphological Variable (tongue + hyoid dataset)**	**PC1** 14.2%	**PC2** 9.4%	**PC3** 8.4%	**PC4** 3.2%
Tongue length/bill length	0.77	0.086	-0.53	-0.11
Bristle proportion	0.66	-0.43	-0.29	0.46
Tongue depth/bill depth	-0.66	0.45	-0.40	0.39
Tongue width/bill width	-0.55	-0.39	-0.69	-0.24
Hyoid length/bill length	-0.29	-0.86	0.20	0.13
