# Supplementary material for: Morphological diversity in the honeyeater hyolingual apparatus and its relationship with nectarivory
Source: PLoS One. 2025 Dec 4;20(12):e0338219. doi: 10.1371/journal.pone.0338219 (PMC12677526; doi:10.1371/journal.pone.0338219)
Supplement: S5 Table — Bolded p-value indicates significance. Significance determined as p < 0.05. (PDF) [file pone.0338219.s008.pdf]

| Variable | AIC | Log Likelihood | $\lambda$ | Slope | Standard Error | t-value | *p*-value |
|---|---|---|---|---|---|---|---|
| Tongue length | 375.43 | -183.72 | 1.02 | 6.82 | 3.3e-5 | 207791 | **0** |
| Bristle length | 297.82 | -144.91 | 1.02 | 5.69 | 1.87 | 3.05 | **0.0034** |
| Tongue depth | 35.26 | -13.63 | 0.97 | 0.11 | 0.25 | 0.46 | 0.65 |
| Tongue width | 71.78 | -31.89 | 0.99 | -0.20 | 0.33 | -0.63 | 0.53 |
| Hyoid length | 257.85 | -124.93 | 0.94 | 4.70 | 7.38 | 0.64 | 0.53 |
