# Supplementary material for: Morphological diversity in the honeyeater hyolingual apparatus and its relationship with nectarivory
Source: PLoS One. 2025 Dec 4;20(12):e0338219. doi: 10.1371/journal.pone.0338219 (PMC12677526; doi:10.1371/journal.pone.0338219)
Supplement: S4 File — S4 Fig (histological cross sections of Melithreptus lunatus) and S5 Fig (microCT scan slices of Ptilotula penicillata) and included and figure legends are provided in the document. (DOCX) [file pone.0338219.s012.docx]

**Methods used to study microscopic tongue anatomy**

We examined the microscopic morphology of the tongue of *Melithreptus lunatus* (UWBM 76699) using histology following the technique used in [1]. We used paraffin histology and implemented the dehydration, embedding, and hematoxylin and eosin-y staining procedures from [2], with the timings modified for the size of our specimen. We then sectioned the tongue at 10-micrometers and viewed and photographed the sections under a Leica DM750 microscope with a Leica EC3 camera attachment at 4X, 10X, and 40X for anatomical description.

The internal structure of the *Ptilotula penicillata* tongue (MCZ Ornithology 364080) was examined using microCT scanning, as it was collected for a separate project examining shape quantification. We used a SkyScan 1173 CT scanner at Museum of Comparative Zoology Digital Imaging Facility at Harvard University, set to 62.5-micron resolution and 40kv, and the tongue was stained with phospho-tungstic acid (PTA) to improve visualization [26]. The 3D reconstruction was done in the software 3D Slicer [3] with the Slicermorph extension [4].

**Descriptive results of microscopic tongue morphology**

Across the Meliphagidae the proximal half of the tongue is composed of a central trough with raised lingual edges. Viewed in cross section, the trough begins as a “U” shape and transitions to a “W” shape moving proximally to distally (Fig S4 and S5). The distal half of the honeyeater tongue can take many different shapes, as described above, but all of those shapes are formed by tissues made of primarily of keratin. The proximal portion of the tongue contains more tissue types and provides the base for the complex shapes formed at the tip. The proximal half of the tongue is composed of many layers of stratum corneum, or keratinized epithelium (Fig S4 B), which are folded inwards at several invagination points (Fig S4 C-D). These invagination points serve as bifurcation points more distally in the tongue when the central groove splits into two or four grooves (Fig S5 A-F). The central core of the tongue contains connective tissue and a cartilaginous rod on either side of the central tongue axis, each of which is dorsoventrally oval shaped in transverse cross sections (Fig S4 C and S5 E-I). Proximally, these rods grow taller and wider as the tongue itself widens (Fig S4 E-F and S5 G-I), providing structural support to the tongue base. These rods contain sinuses, which are likely vascular and provide nutrients and oxygen to the connective tissue in the tongue base (Fig S4 E-F and S5 H-I). These rods and sinuses are found in both *Melithreptus lunatus* and *Ptilotula penicillata* (compare Fig S4 and S5), suggesting that while honeyeaters can have different tongue tip morphologies (*Ptilotula penicillata* is a Type 1 tongue and *Melithreptus lunatus* is a Type 2 tongue), the tongue base is likely to be consistent morphologically at the macro and micro scales.

When comparing the present histological work on *Melithreptus lunatus* with that on *Acanthorhynchus superciliosus* and *Phylidonyris novaehollandiae* [1], there is a notable difference between species in the number of stratum corneum layers and the extent of the invagination of those layers. *Melithreptus lunatus* has a Type 2 tongue shape while *Acanthorhynchus superciliosus* has Type 4 and *Phylidonyris novaehollandiae* has Type 5, but all species show some degree of invagination of the stratum corneum layers, even though the folding is shallow and does not contribute substantially to tongue structure in *Acanthorhynchus superciliosus*. There are more layers of stratum corneum in the tongue walls of *Phylidonyris novaehollandiae* and *Melithreptus lunatus*, while the tongue of *Acanthorhynchus superciliosus* has many fewer layers and is thin walled. The histological images in Collins (2008) show paired cartilaginous rods in *Acanthorhynchus superciliosus* and *Phylidonyris novaehollandiae*, which suggests that those are present in honeyeater tongues regardless of tongue type, however *Acanthorhynchus* is the only genus in which the cartilaginous rods extend to the tongue tip (Fig 1D).

**Literature Cited**

1. Collins BG. Nectar intake and foraging efficiency: responses of honeyeaters and hummingbirds to variations in floral environments. The Auk. 2008;125: 574–587. doi:10.1525/auk.2008.07070

2. Humason GL. Animal tissue techniques. 3rd ed. 3rd ed. San Francisco: W.H. Freemand and Company; 1972.

26. Salmon P. Embryo PTA staining for micro-CT ex-vivo. Bruker microCT; 2012.

3. Kikinis R, Pieper SD, Vosburgh KG. 3D Slicer: A Platform for Subject-Specific Image Analysis, Visualization, and Clinical Support. In: Jolesz FA, editor. Intraoperative Imaging and Image-Guided Therapy. New York, NY: Springer; 2014. pp. 277–289. doi:10.1007/978-1-314-7657-3_19

4. Rolfe S, Pieper S, Porto A, Diamond K, Winchester J, Shan S, et al. SlicerMorph: An open and extensible platform to retrieve, visualize and analyse 3D morphology. Methods in Ecology and Evolution. 2021;12: 1816–1825. doi:10.1111/2041-210X.13669


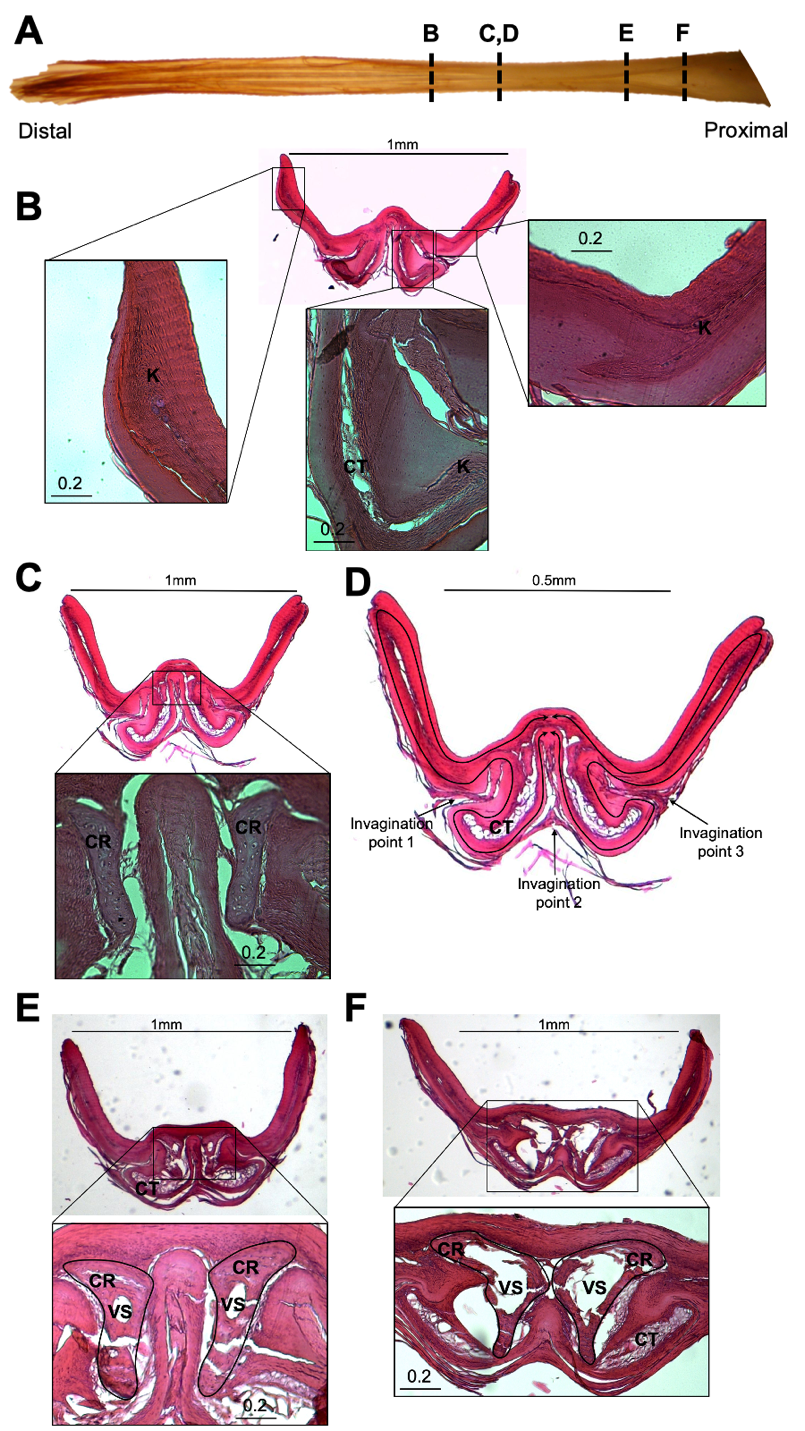


**S4 Fig. Histology of a *Melithreptus lunatus* tongue sectioned in the transverse plane and stained with hematoxylin and eosin.** Panel A shows the location of sections in Panels B-F along the tongue. Panels B-F are arranged proximal to distal and are oriented such that the dorsal side of the tongue is at the top of the image and the ventral side at the bottom. Panel B shows the tongue immediately posterior to the bifurcation into grooves and illustrates the layers of keratin (K) that create the tongue body. Panel C shows the portion of the tongue where cartilaginous supporting rods (CR) become visible and the connective tissue (CT) layer in the center of the tongue thickens. Panel D is a higher magnification image of the section in Panel C, illustrating the invagination points where the keratin layers fold. Panel E shows the portion of the tongue where the vascular sinuses (VS) within the cartilaginous rods (CR) become visible, and where the cartilaginous rods elongate dorsoventrally. Panel F shows the posterior portion of the tongue where the cartilaginous rods (CR) widen to form support for the tongue base and the vascular sinuses (VS) become wider and taller. All sections at 10 microns thick. All scale bars are in mm. Specimen shown is UWBM 76699.


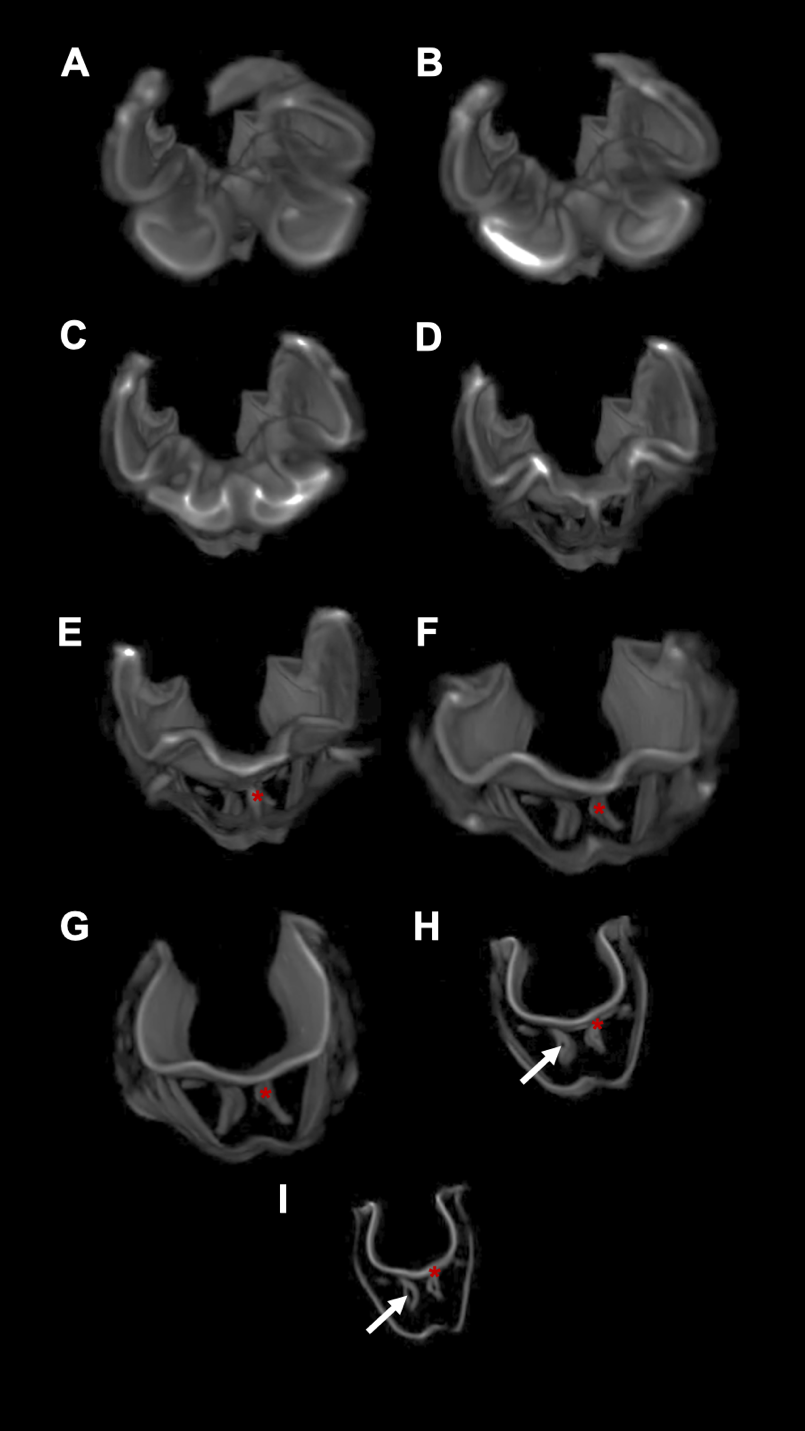


**S5 Fig. Transverse cut aways from 3D reconstruction of a *Ptilotula penicillata* tongue.** Panels A-I are arranged anterior to posterior and are oriented such that the dorsal side of the tongue is at the top of the image. Panels A-C illustrate the four grooves present at the tongue tip. Panel D shows the point immediately posterior to the coalescence of the grooves. Panel E shows the portion of the tongue where cartilaginous supporting rods (red asterisk) become visible. Panels F-G show the tongue widening and the cartilaginous supporting rods (red asterisk) elongate dorsoventrally. Panel H shows the portion of the tongue where the vascular sinuses (white arrow) within the cartilaginous rods (red asterisk) become visible. Panel I shows the posterior portion of the tongue where the vascular sinuses (white arrow) become wider and taller. All panels are at the same scale. Specimen is MCZ Ornithology 364080.
